# CXCL13 Expression Promotes CAR T Cell Antitumor Activity and Potentiates Response to PD‐1 Blockade

**DOI:** 10.1002/advs.202508095

**Published:** 2025-06-10

**Authors:** Yang Zhou, Wenli Zhao, Yihan Zhu, Hongyan Liu, Yicheng Sun, Zhengrong Gong, Xuanyi Li, Ziying Liu, Kang Wen, Yicheng Wang, Jie Ren, Ruipei Xiao, Ling Jiang, Yanfeng Hu, Enguang Bi, Xiaoyong Zhang

**Affiliations:** ^1^ Department of General Surgery Guangdong Provincial Key Laboratory of Precision Medicine for Gastrointestinal Tumor Nanfang Hospital Southern Medical University Guangzhou 510515 China; ^2^ Department of Biochemistry and Molecular Biology School of Basic Medical Sciences Southern Medical University Guangzhou 510515 China; ^3^ Department of Infectious Diseases Nanfang Hospital Southern Medical University Guangzhou 510515 China; ^4^ State Key Laboratory of Organ Failure Research Key Laboratory of Infectious Diseases Research in South China Ministry of Education Guangdong Provincial Key Laboratory for Prevention and Control of Major Liver Diseases Guangdong Provincial Clinical Research Center for Viral Hepatitis Guangdong Institute of Hepatology Guangdong Provincial Research Center for Liver Fibrosis Engineering and Technology Guangzhou 510515 China; ^5^ Department of Urology Zhujiang Hospital Southern Medical University Guangzhou 510282 China; ^6^ Department of Hematology Nanfang Hospital Southern Medical University Guangzhou 510515 China; ^7^ Guangdong Provincial Key Laboratory of Single‐cell and Extracellular Vesicles Southern Medical University Guangzhou 510515 China

**Keywords:** CAR T cells, CXCL13, immune checkpoint blockade

## Abstract

Immune checkpoint blockade (ICB) and chimeric antigen receptor (CAR) T cell therapies have revolutionized cancer immunotherapy, offering significant benefits across various cancers. However, challenges remain, particularly in solid tumors where immunosuppressive tumor microenvironments and T cell exhaustion limit effectiveness. Combining ICB with CAR T cell therapy has shown potential but requires further optimization for effective synergy. Here, the bioinformatic analysis identified that CXCL13 expression is highly elevated in T cells from patients who respond to ICB, indicating its possible role in enhancing T cell antitumor responses. Mouse CAR T cells are engineered to overexpress CXCL13 and observed that these cells displayed reduced exhaustion, increased central memory phenotype, and improved mitochondrial function and proliferation in an AKT‐mTOR dependent manner. CXCL13‐overexpressing CAR T cells show significantly increased antitumor activity in vivo, particularly when combined with PD‐1 inhibition, promoting the expansion and persistence of early exhausted CD8^+^ CAR T cells. CXCL13 also conferred similar in vitro phenotypic enhancements in human CAR T cells as observed in murine cells. These results indicate that CXCL13 expression improves CAR T cell function and responsiveness to ICB, offering a promising and translationally relevant strategy to optimize CAR T cell therapy for solid tumors in clinical settings.

## Graphical abstract

This study demonstrates that engineering CAR T cells to express CXCL13 enhances their antitumor efficacy and significantly improves responsiveness to PD‐1 immune checkpoint blockade. CXCL13 promotes T cell persistence, and resistance to early exhaustion via the AKT‐mTOR pathway. These findings suggest a promising strategy to overcome key barriers in CAR T cell therapy for solid tumors.

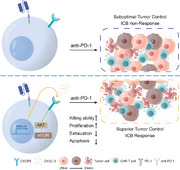

## 1 Introduction

T cells are pivotal in tumor immunotherapy, with ICB therapy and adoptive cell therapy (ACT) standing as two principal approaches.^[^ ^1^, ^2^ ^]^ ICB therapy, particularly PD‐1 blockade, counteracts T cell exhaustion by targeting the inhibitory PD‐1 molecule on T cells, restoring their functionality and achieving significant success in several cancers, such as non‐small cell lung cancer (NSCLC) and melanoma.^[^ ^3^, ^4^ ^]^ Despite these achievements, response rates remain suboptimal across diverse patient populations.^[^ ^5^ ^]^ In immunosuppressive tumor microenvironment (TME), limited tumor infiltration and inefficient presentation of tumor‐associated antigens restrict the abundance of tumor‐specific T cells, consequently reducing the effectiveness of PD‐1 blockade.^[^ ^5^ ^]^ As a parallel approach, ACT—particularly chimeric antigen receptor T cell (CAR T) therapy—enables T cells to recognize tumor antigens independently of MHC restrictions, providing a robust pool of tumor‐targeted T cells.^[^ ^6^ ^]^ While CAR T therapy has shown remarkable success in hematological cancers, its efficacy in solid tumors is hindered by immunosuppressive TME and the onset of CAR T cell exhaustion after prolonged antigen exposure.^[^ ^7^, ^8^, ^9^, ^10^ ^]^ Combining CAR T therapy with PD‐1 blockade has potential synergistic effects that could overcome these limitations, making it a key focus of tumor immunotherapy research.

Although the combination of CAR T and PD‐1 blockade has shown promise in hematologic cancers,^[^ ^11^, ^12^ ^]^ its efficacy in solid tumors remains limited. In a phase I trial with neuroblastoma patients,^[^ ^13^ ^]^ PD‐1 inhibition did not significantly improve CAR T cell expansion or persistence, and similar limitations were observed in glioblastoma.^[^ ^14^ ^]^ Additionally, a phase I study of mesothelin‐targeting CAR T cells with PD‐1 and TCR disruption in solid tumors showed limited efficacy, with only stable disease achieved in less than 15% of patients.^[^ ^15^ ^]^ These suboptimal responses are likely attributed to the TME's heterogeneity and immunosuppressive nature. Enhancing CAR T cell responsiveness to PD‐1 blockade is thus critical for improving the efficacy of combination therapies in solid tumors.

CXCL13^+^ T cells are associated with favorable responses to ICB therapy.^[^ ^16^, ^17^, ^18^ ^]^ However, their application in ACT is constrained due to their low frequency in the TME, complicating isolation and expansion, with no established protocols for direct differentiation in vitro. In this study, we aimed to address these challenges by overexpressing CXCL13 in CAR T cells. This approach enabled us to assess the impact of CXCL13 on CAR T cell antitumor activity and its potential to enhance therapeutic efficacy both as a standalone therapy and in combination with ICB. Our findings indicate that CXCL13‐overexpression enhances CAR T cell antitumor efficacy and responsiveness to PD‐1 blockade, suggesting a promising strategy for advancing CAR T therapy in solid tumors.

## 2 Results

### 2.1 Elevated CXCL13 Expression in T Cells from Responders in ICB Therapy

**Figure 1:**
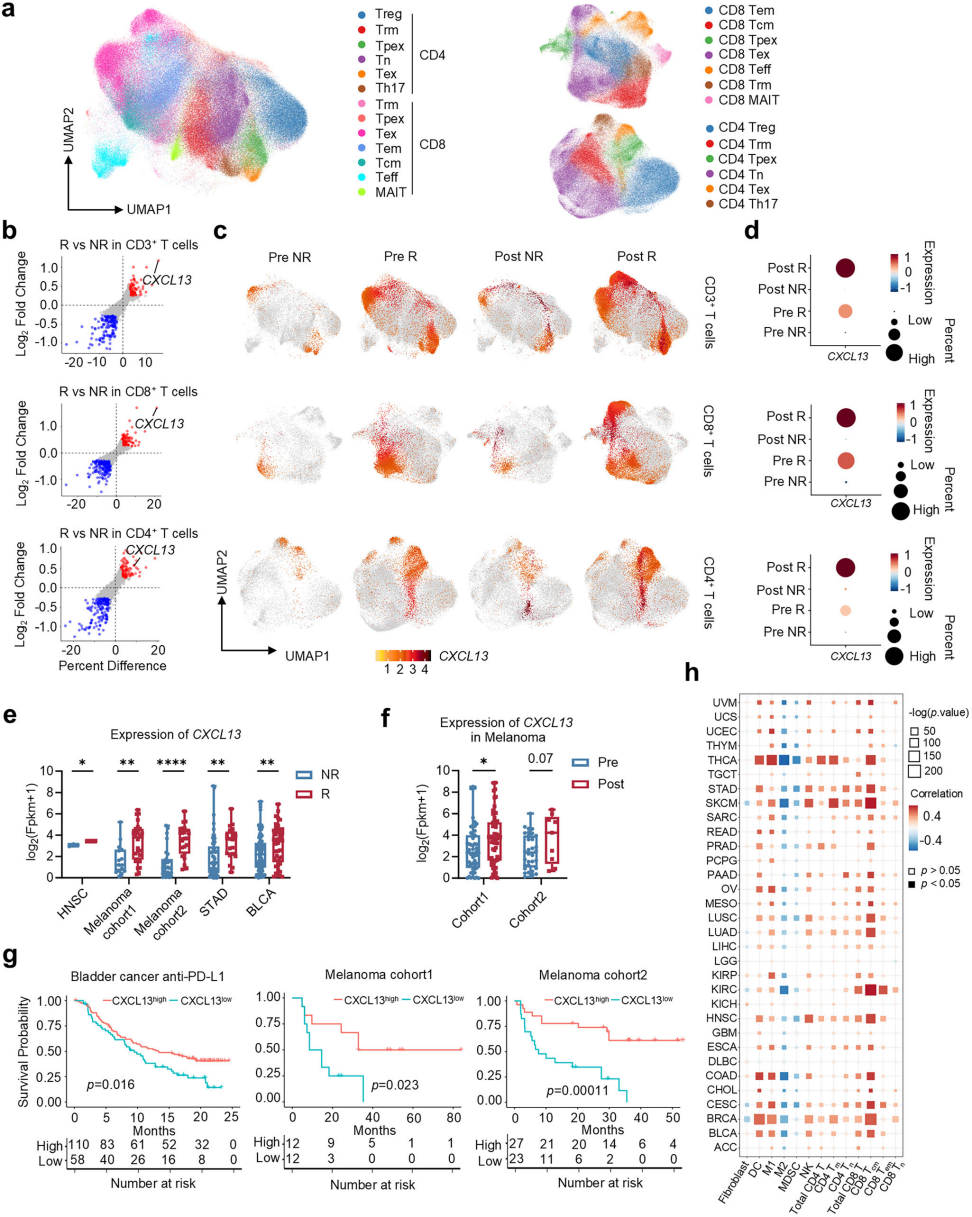
*CXCL13* is highly expressed in T cells from responders to ICB therapy. a). UMAP plot displaying 237231 CD3^+^ T cells across 8 cancer types: BCC (basal cell carcinoma, *n* = 11 patients; *n* = 22 samples), SCC (squamous cell carcinoma, *n* = 3 patients; *n* = 8 samples), BLCA (bladder cancer, *n* = 6 patients; *n* = 6 samples), TNBC (triple‐negative breast cancer, *n* = 8 patients; *n* = 14 samples), RCC (Renal cell carcinoma, *n* = 7 patients; *n* = 7 samples), HNSCC (head and neck cancer, *n* = 4 patients; *n* = 8 samples), NSCLC (non‐small‐cell lung cancer, *n* = 36 patients; *n* = 47 samples), and PRAD (prostate cancer, *n* = 10 patients; *n* = 12 samples), categorized into 13 distinct cell types. b). Comparison of differential genes in CD3^+^, CD8^+^, and CD4^+^ T cells in the response and non‐response groups. c,d). UMAP plots (c) and dot plots (d) showing the expression of *CXCL13* in CD3^+^, CD8^+^, and CD4^+^ T cells of pre‐treatment non‐response, pre‐treatment response, post‐treatment non‐response, and post‐treatment response groups. e). *CXCL13* expression levels in responders and non‐responders across multiple tumor cohorts treated with ICB: HNSC, melanoma cohort 1 (anti‐PD‐1 and anti‐CTLA‐4), melanoma cohort 2 (anti‐PD‐1), STAD (stomach adenocarcinoma), and BLCA. f). Comparison of *CXCL13* expression in pre‐treatment and post‐treatment groups from melanoma cohort 1 and melanoma cohort 2 treated with ICB. g). Kaplan‐Meier survival curves stratified by high and low *CXCL13* expression in ICB‐treated patients from BLCA, melanoma cohort 1, and melanoma cohort 2. h). Heatmap showing the correlation of *CXCL13* expression with various cell types across tumor types in the TCGA dataset. Cell‐type deconvolution was performed using TIMER2.0, with significant correlations (*p* < 0.05) marked by solid indicators.

To identify key genes driving T cell responses in cancer immunotherapy, we collected single‐cell RNA sequencing (scRNA‐seq) data and bulk RNA‐seq data from patients who received ICB therapy (Figure S1, Supporting Information). We analyzed scRNA‐seq data from 8 tumors in ICB therapy, including BLCA,^[^ ^19^ ^]^ HNSCC,^[^ ^20^ ^]^ TNBC,^[^ ^21^ ^]^ NSCLC,^[^ ^22^ ^]^ PRAD,^[^ ^23^ ^]^ RCC,^[^ ^24^ ^]^ BCC and SCC data,^[^ ^25^ ^]^ and obtained 237231 high‐quality CD3^+^ T cells, including 85 patients and 124 samples (Supplementary Table 1). Clustering analysis identified two major T cell subsets: CD8^+^ and CD4^+^ T cells, including 13 cell groups (**Figure** **1**a). *CXCL13* was identified as a top upregulated gene in CD3^+^, CD8^+^, and CD4^+^ T cells from responders by differential expression analysis between responders and non‐responders (Figure 1b). This finding was further supported by the feature plots and dot plots, which revealed increased *CXCL13* expression in CD3^+^, CD4^+^, and CD8^+^ T cells from responders (Figure 1c,d). Then we carried out the sample‐level analysis, and T cells were divided into two subgroups based on the scaled expression of *CXCL13*: *CXCL13* ^+^ cells (scaled expression of *CXCL13* > 0) and *CXCL13* ^−^ cells (scaled expression of *CXCL13* < 0) (Figure S2a–c, Supporting Information). We discovered that the percentage of *CXCL13* ^+^ cells was higher in the responder group and increased after therapy (Figure S2d–i, Supporting Information). Subset‐specific analysis further revealed that *CXCL13* expression was predominantly restricted to Tex and Tpex cells within both CD4⁺ and CD8⁺ compartments (Figure S3a,b, Supporting Information). Gene Set Enrichment Analysis (GSEA) analysis comparing *CXCL13*⁺ versus *CXCL13* ^−^ cells within the CD4⁺ Tex and CD8⁺ Tex populations demonstrated that pathways related to leukocyte chemotaxis were selectively enriched in the *CXCL13*⁺ subset (Figure S3c,d, Supporting Information). Notably, tumor‐reactive gene signatures^[^ ^26^ ^]^ were enriched in *CXCL13*⁺ Tex cells, whereas non‐tumor‐reactive pathways were preferentially enriched in *CXCL13*⁻ Tex cells (Figure S3e,f, Supporting Information). Together, these findings highlight CXCL13 as a potential marker of tumor‐reactive, migratory, and functionally poised T cell subsets that may contribute to favorable clinical responses following ICB therapy. To confirm the association between *CXCL13* expression and response to ICB treatments, we expanded the analysis to larger ICB cohorts,^[^ ^27^, ^28^, ^29^, ^30^ ^]^ observing consistently elevated *CXCL13* levels in responders and post‐treatment samples (Figure 1e,f). Additionally, *CXCL13* expression correlated positively with improved survival outcomes in both ICB and TCGA cohorts (Figure 1g; Figure S4, Supporting Information). Further analysis demonstrated that CXCL13 is primarily secreted by T cells and acts on B cells and T cells through CXCR5 (Figure S5a,b, Supporting Information). Intercellular communication sourced from CD4^+^ T cells and CD8^+^ T cells mainly targets T cells (Figure S5c,d, Supporting Information). Using TIMER2.0,^[^ ^31^ ^]^ cell type deconvolution analysis revealed that *CXCL13* expression positively correlated with immune cell subsets known to promote antitumor immunity (e.g., DC cells, M1 macrophages, NK cells, and CD4^+^ and CD8^+^ T cell subsets) and inversely correlated with immunosuppressive subsets (e.g., cancer‐associated fibroblasts, M2 macrophages, and MDSCs) across cancers (Figure 1h).

These results indicate that *CXCL13* ^+^ T cells are associated with a favorable response to ICB therapy, suggesting that increasing *CXCL13* ^+^ T cell populations could enhance ICB therapy efficacy in cancer immunotherapy.

### 2.2 Enhanced Antitumor Activity in CXCL13‐Overexpressing CAR T Cells

**Figure 2:**
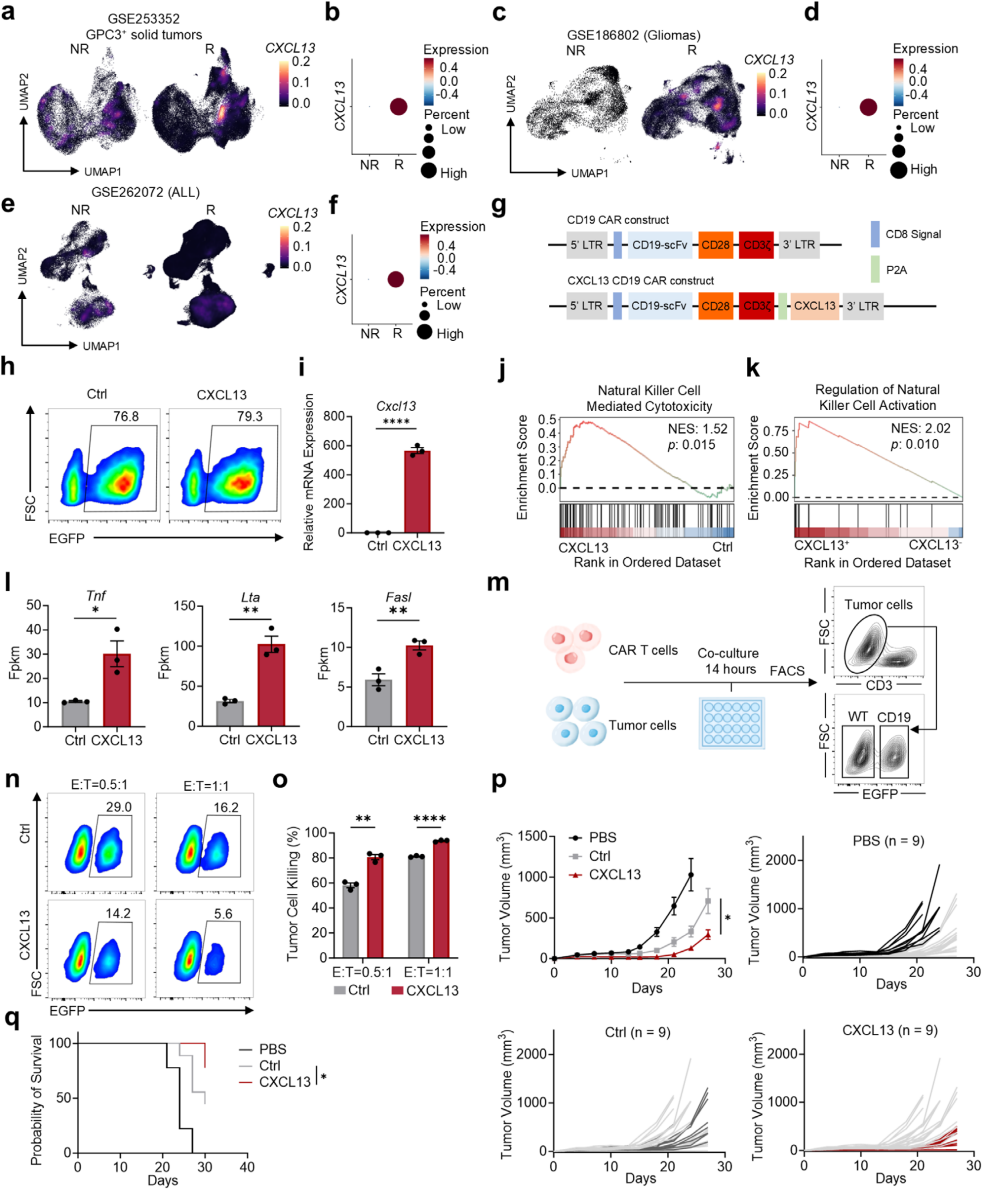
CXCL13‐overexpression enhances the antitumor activity of CAR T cells both in vitro and in vivo. a,b). UMAP plots (a) and dot plots (b) of T cell populations from patients with GPC3‐positive solid tumors treated with CAR T cells, stratified into non‐responders (NR, *n* = 2 patients, *n* = 6 samples) and responders (R, *n* = 5 patients, *n* = 15 samples). c,d). UMAP plots (c) and dot plots (d) of T cell populations from patients with gliomas treated with CAR T cells (NR, *n* = 1 patient, *n* = 5 samples; R, *n* = 3 patients, *n* = 18 samples). e,f). UMAP plots (e) and dot plots (f) of T cell populations from patients with acute lymphocytic leukemia (ALL) treated with CAR T cells (NR, *n* = 6 patients, *n* = 6 samples; R, *n* = 76 patients, *n* = 76 samples). g). Schematic representation of the structures of the CD19 CAR and CXCL13 CD19 CAR constructs. h). Percentage of EGFP^+^ cells in CAR T cells and CXCL13 CAR T cells, indicating CD19 CAR expression. i. mRNA expression levels of Cxcl13 in control CAR T cells and CXCL13 CAR T cells. Mean ± SEM, *n* = 3. j,k). GSEA analysis of the natural killer cell gene set in CXCL13 CAR T cells versus control CAR T cells using bulk RNA‐seq data (j) and *CXCL13* ^+^ cells versus *CXCL13* ^−^ cells using scRNA‐seq data (k). l). Expression levels of *Tnf*, *Lta*, and *Fasl* in control CAR T cells and CXCL13 CAR T cells. Mean ± SEM, n = 3. m). Flowchart of CAR T cell‐mediated tumor cell killing assay. CAR T cells were co‐cultured with a 1:1 mixture of MC38 WT (EGFP^−^) and MC38‐CD19 (EGFP^+^) tumor cells for 14 h at different effector‐to‐target (E:T) ratios, and the percentage of EGFP^+^ live tumor cells was measured. n). Percentage of EGFP^+^ tumor cells in the control CAR T and CXCL13 CAR T groups. o. Killing efficiency of CAR T cells and CXCL13 CAR T cells. Mean ± SEM, *n* = 3. p,q). In vivo treatment of subcutaneous B16‐CD19 tumors. Tumor‐bearing mice were treated with 2×10⁶ CAR T cells on day 5 post‐tumor establishment. (p). Tumor growth represented as mean tumor size (*n* = 9 per group: PBS, CAR T, CXCL13 CAR T). Mean ± SEM. (q). Survival of B16‐CD19 tumor‐bearing mice treated with CAR T cells (*n* = 9 per group: PBS, CAR T, CXCL13 CAR T). ^*^ *p* < 0.05, ^**^ *p* < 0.01, ^***^ *p* < 0.001, ^****^ *p* < 0.0001; two‐tailed unpaired t‐test (i,l,o,p); log‐rank (Mantel‐Cox) test (q).

Since there is no established strategy to differentiate *CXCL13* ^+^ T cells in vitro and in vivo, we wonder whether overexpression of *CXCL13* in T cells can enhance the therapeutic efficacy of T cells, especially in CAR T cells, which is the most important strategy in ACT. Firstly, we found *CXCL13* is highly expressed in T cells from responders of CAR T treated solid^[^ ^32^, ^33^ ^]^ and hematological tumors^[^ ^34^ ^]^ (**Figure** **2**a–f), which potentiates the possibility. To investigate whether CXCL13‐overexpression could enhance CAR T cell function, we generated CD19‐targeted CAR T cells engineered to express mouse CXCL13 via a tandem construction with a cleavable P2A peptide (Figure 2g). CAR transfection efficiency in CXCL13‐overexpressing CAR T cells was similar to that of control CAR T cells (Figure 2h), but CXCL13 CAR T cells showed significantly higher *Cxcl13* expression (Figure 2i). To investigate how CXCL13 operates on CAR T cells, we conducted a bulk RNA‐seq analysis, and GSEA revealed similar enhanced cytotoxic potential in CXCL13 CAR T cells in bulk RNA‐seq (Figure 2j) and *CXCL13* ^+^ T cells in scRNA‐seq (Figure 2k), with upregulation of cytotoxicity‐related genes (*Tnf*, *Lta*, *Fasl*) (Figure 2l). In vitro co‐culture assays with tumor cells demonstrated that CXCL13 CAR T cells had significantly greater tumor‐killing efficacy compared to control CAR T cells (Figure 2m–o). Furthermore, we investigated their anti‐tumor potential in vivo, and studies using B16‐CD19 tumor‐bearing mice confirmed that CXCL13‐overexpression significantly enhanced CAR T cell‐mediated antitumor activity (Figure 2p), with a prolonged survival period (Figure 2q).

Given that CXCL13 is known to recruit multiple immune cell types, including T cells, macrophages, and B cells, we next investigated whether CXCL13 CAR T cells could enhance endogenous immune cell infiltration within tumors. Specifically, we examined the abundance of endogenous CD4⁺ and CD8⁺ T cells, as well as macrophages, in tumors treated with CXCL13 CAR T cells. Flow cytometry analysis revealed minimal changes in these populations compared to tumors treated with control CAR T cells (Figure S6a–c, Supporting Information). As anticipated, B cells were undetectable in the peripheral blood of mice receiving either control or CXCL13 CAR T cells, due to the CD19‐targeting design of the CAR construct (Figure S6d, Supporting Information).

To assess whether CXCL13 might recruit B cells into the tumor—given their essential role in tertiary lymphoid structure (TLS) formation^[^ ^35^ ^]^—we generated a new CXCL13‐overexpressing CAR T cell targeting Claudin 18.2 (CLDN18.2), thereby preserving endogenous B cells (Figure S6e,Supporting Information). In the B16‐CLDN18.2 tumor model, CXCL13 CLDN18.2 CAR T cells exhibited superior antitumor activity (Figure S6f, Supporting Information). Consistent with our previous findings, the frequencies of intratumoral CD4⁺ and CD8⁺ T cells remained largely unchanged (Figure S6g, Supporting Information). However, we observed a marked increase in B cell infiltration in tumors treated with CXCL13 CLDN18.2 CAR T cells (Figure S6h, Supporting Information), supporting the role of CXCL13 in facilitating B cell recruitment into the TME.

To further investigate potential interactions between CXCL13 CAR T cells and endogenous immune cells, we employed the MB49‐CD19 bladder tumor model, which is known for heightened immune infiltration. As expected, CXCL13 CAR T cells demonstrated superior tumor control in this model (Figure S6i, Supporting Information). Immunofluorescence staining of tumor sections for CD3, CD45.1, and F4/80 revealed no significant differences in the distribution or abundance of T cells, transferred CAR T cells, or macrophages (Figure S6j, Supporting Information), corroborating our flow cytometry data. Collectively, these findings suggest that while CXCL13 may selectively enhance B cell recruitment under conditions that preserve B cell populations, its influence on endogenous T cells and macrophages appears limited in the tumor models tested.

### 2.3 Reduced Exhaustion and Enhanced Central Memory Status in CXCL13 CAR T Cells

**Figure 3:**
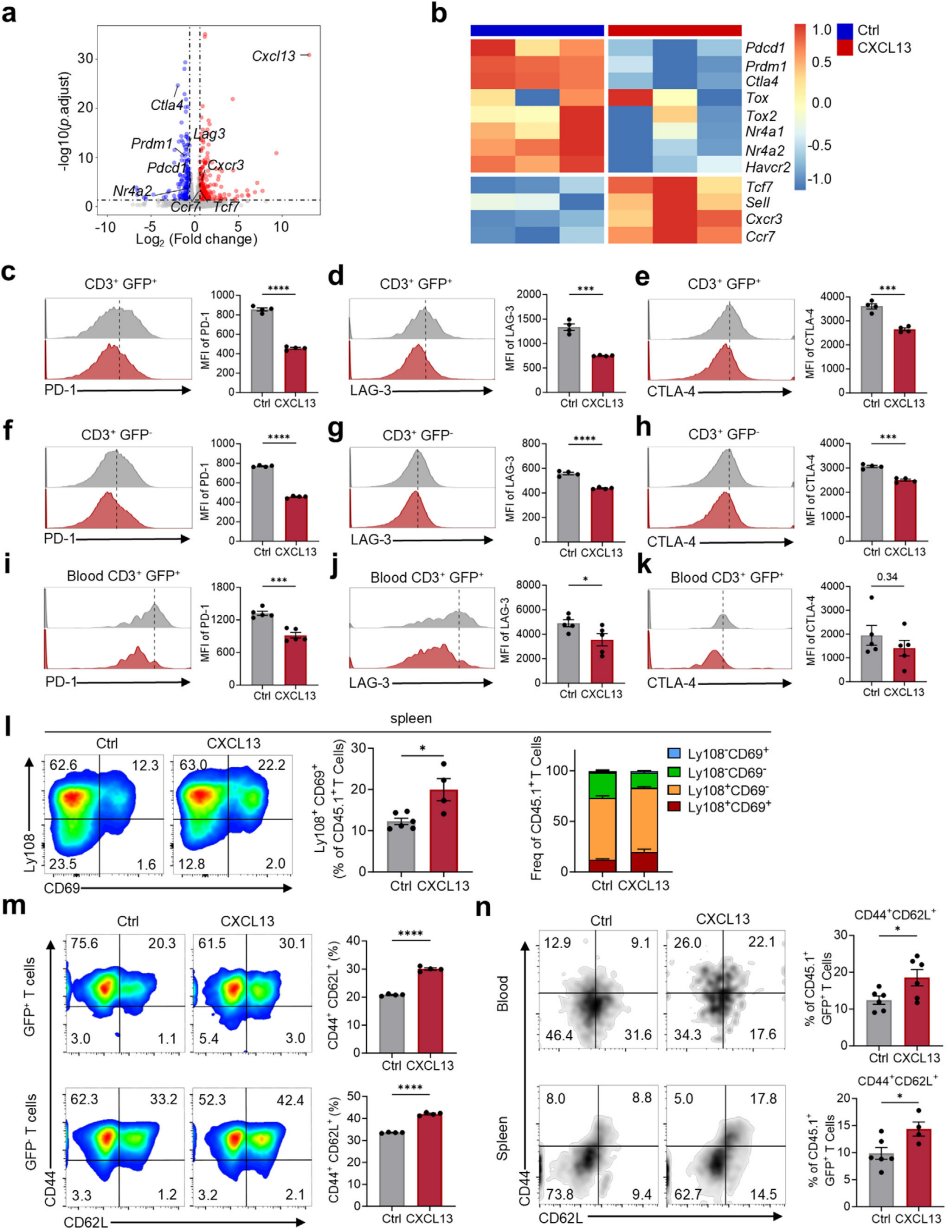
CXCL13 CAR T cells possess reduced exhaustion and enhanced central memory characteristics. a). Volcano plot comparing gene expression profiles between control CAR T cells and CXCL13 CAR T cells. b). Heatmap displaying stem‐like property‐ and exhaustion‐related gene expression in control CAR T cells and CXCL13 CAR T cells. c–h). In vitro expression levels of exhaustion‐related proteins, including PD‐1 (c, f), LAG‐3 (d,g), and CTLA‐4 (e,h), in control CAR T cells and CXCL13 CAR T cells. Mean ± SEM, *n* = 4. i–k). Expression of exhaustion‐related proteins in blood samples from control CAR T cell‐ and CXCL13 CAR T cell‐treated groups, including PD‐1 (i), LAG‐3 (j), and CTLA‐4 (k). Mean ± SEM, *n* = 5. l. Expression of Ly108 and CD69 in CAR T cells isolated from the spleen. Mean ± SEM, *n* = 6 (control CAR T), *n* = 4 (CXCL13 CAR T). m). Expression of CD44 and CD62L in CAR T cells and CXCL13 CAR T cells cultured in vitro for 7 days. Mean ± SEM, *n* = 4. n). Expression of CD44 and CD62L in CAR T cells from blood (*n* = 6) and spleen (*n* = 6 for control CAR T; *n* = 4 for CXCL13 CAR T). Mean ± SEM. ^*^ *p* < 0.05, ^**^ *p* < 0.01, ^***^ *p* < 0.001, ^****^ *p* < 0.0001; two‐tailed unpaired t‐test (c–n).

Further RNA‐seq analysis revealed that CXCL13 CAR T cells showed increased expression of stem‐like property‐associated genes and decreased expression of exhaustion‐related genes (**Figure** **3**a,b). Flow cytometry analysis confirmed reduced levels of exhaustion markers (PD‐1, LAG‐3, and CTLA‐4) in CXCL13 CAR T cells both in vitro (Figure 3c–h; Figure S7a–l, Supporting Information) and post‐infusion in vivo (Figure 3i–k).

Using a four‐stage T cell exhaustion model defined by CD69 and Ly108 expression—progenitor 1 (Ly108^+^CD69^+^; Texprog1), progenitor 2 (Ly108^+^CD69^−^; Texprog2), intermediate (Ly108^−^CD69^−^; Texint), and terminal (Ly108^−^CD69^+^; Texterm)^[^ ^36^ ^]^—we found that CXCL13 CAR T cells contained a higher proportion of Texprog1 cells (Figure 3l; Figure S7m,n, Supporting Information), indicating a less exhausted phenotype. Since a central memory phenotype is positively correlated with improved therapeutic efficacy in CAR T cells, we further assessed the expression of memory markers CD44 and CD62L. CXCL13 CAR T cells demonstrated an increased proportion of CD44^+^CD62L^+^ central memory T cells (TCM) both in vitro (Figure 3m) and after transfer in vivo (Figure 3n).

These findings indicate that CXCL13 CAR T cells exhibit reduced exhaustion and an elevated central memory phenotype, collectively contributing to enhanced proliferative potential and sustained antitumor efficacy.

### 2.4 Enhanced Viability and Proliferation in CXCL13 CAR T Cells

**Figure 4:**
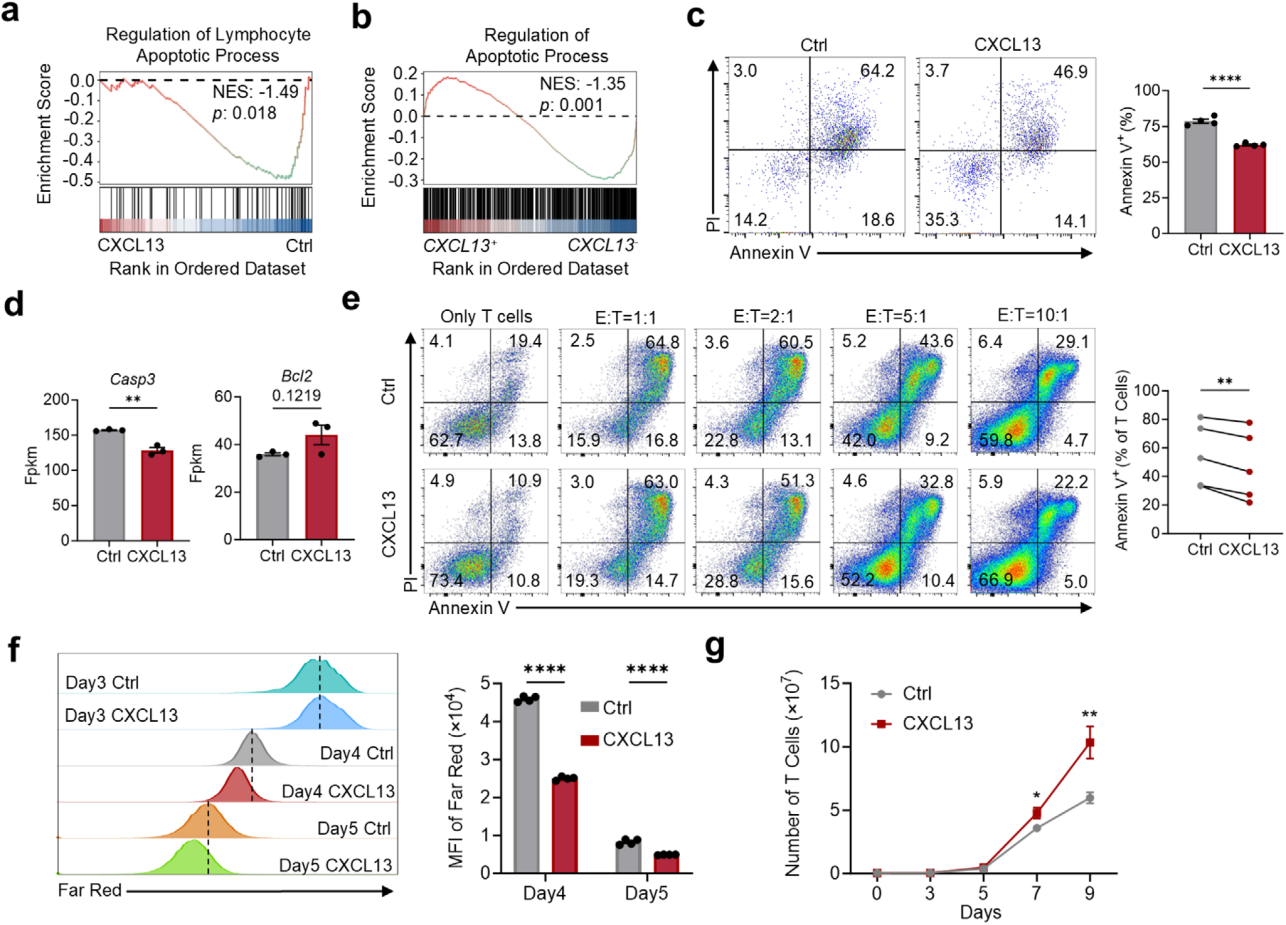
CXCL13 CAR T cells exhibit reduced apoptosis and enhanced proliferation. a,b). GSEA analysis of apoptosis‐related gene sets in bulk RNA‐seq data comparing CXCL13 CAR T cells and control CAR T cells (a) and in scRNA‐seq data comparing *CXCL13* ^+^ cells and *CXCL13* ^−^ cells (b). c). Apoptosis levels in control CAR T cells and CXCL13 CAR T cells cultured alone in vitro on day 10. Mean ± SEM, *n* = 4. d). Expression levels of apoptosis‐related genes (*Casp3* and *Bcl2*) in CAR T cells. Mean ± SEM, *n* = 3. e). Apoptosis levels in control CAR T cells and CXCL13 CAR T cells co‐cultured with tumor cells (MC38 WT [EGFP^−^]: MC38‐CD19 [EGFP^+^] = 1:1) at different T cell‐to‐tumor cell ratios (E:T). Mean ± SEM, *n* = 5. f). Proliferation of CAR T cells was assessed by Far Red staining on day 3, with fluorescence intensity measured on days 4 and 5. Mean ± SEM, *n* = 4. g). Total number of CAR T cells from day 0 to day 9. Mean ± SEM, *n* = 6. ^*^ *p* < 0.05, ^**^ *p* < 0.01, ^***^ *p* < 0.001, ^****^ *p* < 0.0001; two‐tailed unpaired t‐test (c,d,f,g), two‐tailed paired t‐test (e).

Sustained viability and reduced apoptosis are essential for the persistence and effectiveness of CAR T cells. GSEA revealed similar less apoptosis in CXCL13 CAR T cells in bulk RNA‐seq (**Figure** **4**a) and *CXCL13* ^+^ T cells in scRNA‐seq (Figure 4b). Apoptosis assay demonstrated that CXCL13 CAR T cells exhibited significantly improved viability during long‐term culture, with lower apoptosis levels (Figure 4c), reduced expression of the pro‐apoptotic gene *Casp3*, and elevated expression of the anti‐apoptotic gene *Bcl2* (Figure 4d). During tumor targeting, CAR T cells undergo strong stimulation that can trigger activation‐induced cell death (AICD), which contributes to their reduced persistence.^[^ ^37^, ^38^ ^]^ To assess the effect of AICD, we co‐cultured CAR T cells with tumor cells at various ratios and observed that CXCL13 CAR T cells consistently showed reduced apoptosis (Figure 4e).

We further investigated the proliferation of CAR T cells using Far Red staining, which revealed that CXCL13 CAR T cells exhibited a markedly higher proliferative potential, leading to an increase in cell number in vitro (Figure 4f,g). The enhanced viability and proliferation of CXCL13 CAR T cells are closely associated with their enhanced antitumor efficacy.

### 2.5 Antitumor‐Favoring Characteristics in CXCL13 CAR T Cells

**Figure 5:**
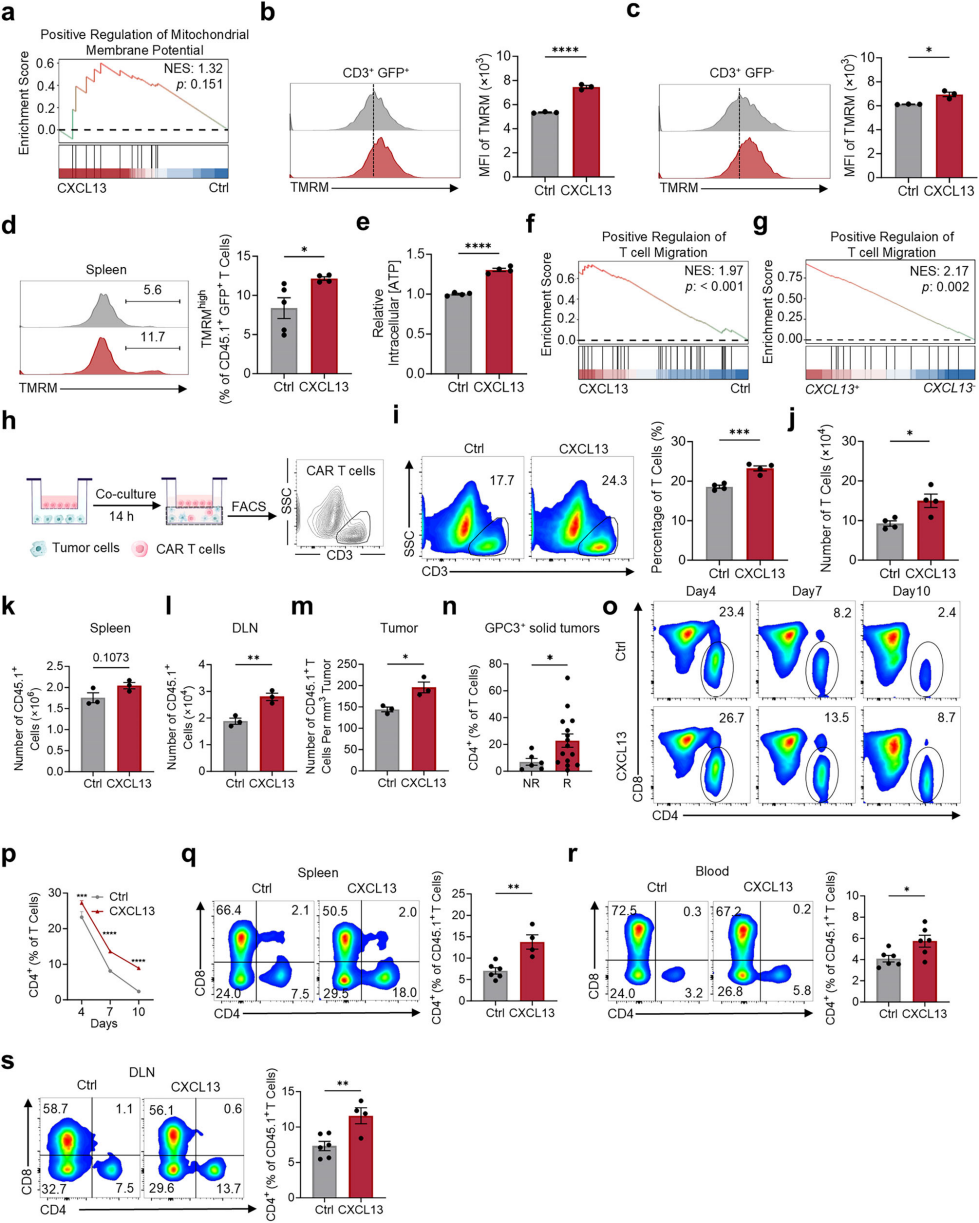
CXCL13 CAR T cells display enhanced antitumor characteristics. a). GSEA analysis of the mitochondrial membrane potential gene set in bulk RNA‐seq comparing CXCL13 CAR T cells with control CAR T cells. b–d). Flow cytometry analysis of mitochondrial membrane potential (TMRM) in control CAR T and CXCL13 CAR T cells. Representative TMRM peak plots are shown for GFP^+^ (b) and GFP^−^ (c) T cells cultured in vitro on day 10 and for cells isolated from the spleen (d). TMRM: Tetramethylrhodamine, methyl ester. Mean ± SEM, *n* = 3 (b, c); *n* = 5 (control CAR T), *n* = 4 (CXCL13 CAR T) (d). e). Intracellular ATP levels in control CAR T cells and CXCL13 CAR T cells. Mean ± SEM, *n* = 4. f,g). GSEA analysis of positive regulation of T cell migration gene set in CXCL13 CAR T cells versus control CAR T cells using bulk RNA‐seq data (f) and *CXCL13* ^+^ cells versus *CXCL13* ^−^ cells using scRNA‐seq data (g). h). Schematic overview of the transwell migration assay. i,j). The migration ability of CAR T cells was assessed by the transwell assay, showing the proportion (i) and number (j) of cells that migrated through the membrane. Mean ± SEM, *n* = 4. k–m). C57BL/6 mice injected s.c. with 5 × 10^5^ B16‐CD19 cells were treated with 2×10⁶ CAR T cells on day 5 post‐tumor establishment. The number of CAR T cells was detected after 7 days of adoptive transfer in the spleen (k), draining lymph nodes (l), and tumor sites (m). Mean ± SEM, *n* = 3. n). Percentage of CD4^+^ T cells among CD3^+^ T cells from patients with GPC3‐positive solid tumors treated with CAR T cells (non‐responders [NR], *n* = 6; responders [R], *n* = 15). Mean ± SEM. o,p). Flow cytometry plots (o) and line graphs (p) showing the percentage of CD4⁺ CAR T cells in vitro from day 4 to day 10. Mean ± SEM, *n* = 4. q–s). Proportions of CD4^+^ and CD8^+^ CAR T cells in blood (q), spleen (r), and draining lymph nodes (s). Mean ± SEM, blood (*n* = 6), spleen (*n* = 6 for control CAR T; *n* = 4 for CXCL13 CAR T), draining lymph nodes (*n* = 6 for control CAR T; *n* = 4 for CXCL13 CAR T). ^*^ *p* < 0.05, ^**^ *p* < 0.01, ^***^ *p* < 0.001, ^****^ *p* < 0.0001; two‐tailed unpaired t‐test (b–e,i–n,p–s).

Given the critical role of mitochondrial health in T cell antitumor efficacy,^[^ ^39^ ^]^ GSEA indicated an upregulation trend in the mitochondrial protein pathway of CXCL13 CAR T cells (**Figure** **5**a), and we assessed mitochondrial function in CXCL13 CAR T cells and observed a higher mitochondrial membrane potential both in vitro and in vivo (Figure 5b–d) and increased ATP production (Figure 5e), indicative of superior mitochondrial function. Solid tumors are dense tissues, and one major factor limiting the effectiveness of CAR T cell therapy in solid tumors is the difficulty for CAR T cells to migrate into the tumor tissue.^[^ ^40^ ^]^ Therefore, enhancing the migratory capacity of CAR T cells is crucial for improving therapeutic outcomes in solid tumors. GSEA revealed similar enhanced T cell migration in CXCL13 CAR T cells in bulk RNA‐seq (Figure 5f) and *CXCL13* ^+^ T cells in scRNA‐seq (Figure 5g). Using a transwell assay, we evaluated the migration capacity of CAR T cells and found that CXCL13 CAR T cells demonstrated superior migration potential (Figure 5h–j), indicating a stronger ability to combat solid tumors. To assess the impact of CXCL13 on in vivo CAR T cell migration, we analyzed the distribution of CAR T cells in tumor‐bearing mice seven days after adoptive transfer. While CXCL13‐overexpression did not significantly alter CAR T cell accumulation in the spleen (Figure 5k), it markedly enhanced CAR T cell infiltration into both the tumor‐draining lymph nodes (Figure 5l) and tumor sites (Figure 5m). However, when evaluating the proportion of CD45.1⁺ CAR T cells among total viable cells, no significant difference was observed between groups (Figure S8a, Supporting Information). This discrepancy is likely due to reduced tumor necrosis and higher viable cell density in the CXCL13 group, resulting in increased absolute CAR T cell numbers without a corresponding change in their proportional representation among live cells. In a clinical trial of CAR T therapy for multiple myeloma, a higher CD4^+^/CD3^+^ ratio was associated with improved clinical outcomes.^[^ ^41^ ^]^ In CAR T therapy for solid tumors, we also found that a higher CD4^+^/CD3^+^ ratio was associated with better therapeutic outcomes by analyzing public scRNA‐seq data^[^ ^33^, ^42^ ^]^ (Figure 5n; Figure S8b, Supporting Information). Therefore, we assessed the CD4^+^/CD3^+^ ratio of CAR T cells both in vitro and in vivo, and found that CXCL13 CAR T cells had a higher CD4^+^/CD3^+^ ratio (Figure 5o–s). Moreover, EGFP, which indicates CAR expression, is often lost during long‐term culture, and we observed that CXCL13 CAR T cells maintained a higher proportion of EGFP expression (Figure S8c, Supporting Information).

The data above demonstrated that CXCL13‐expressing CAR T cells possess multiple characteristics to facilitate their tumor immunotherapeutic efficacy.

### 2.6 The CXCL13‐CXCR5‐AKT/mTOR Axis Enhances CAR T Cell Cytotoxicity and Proliferation While Suppressing Exhaustion and Apoptosis

**Figure 6:**
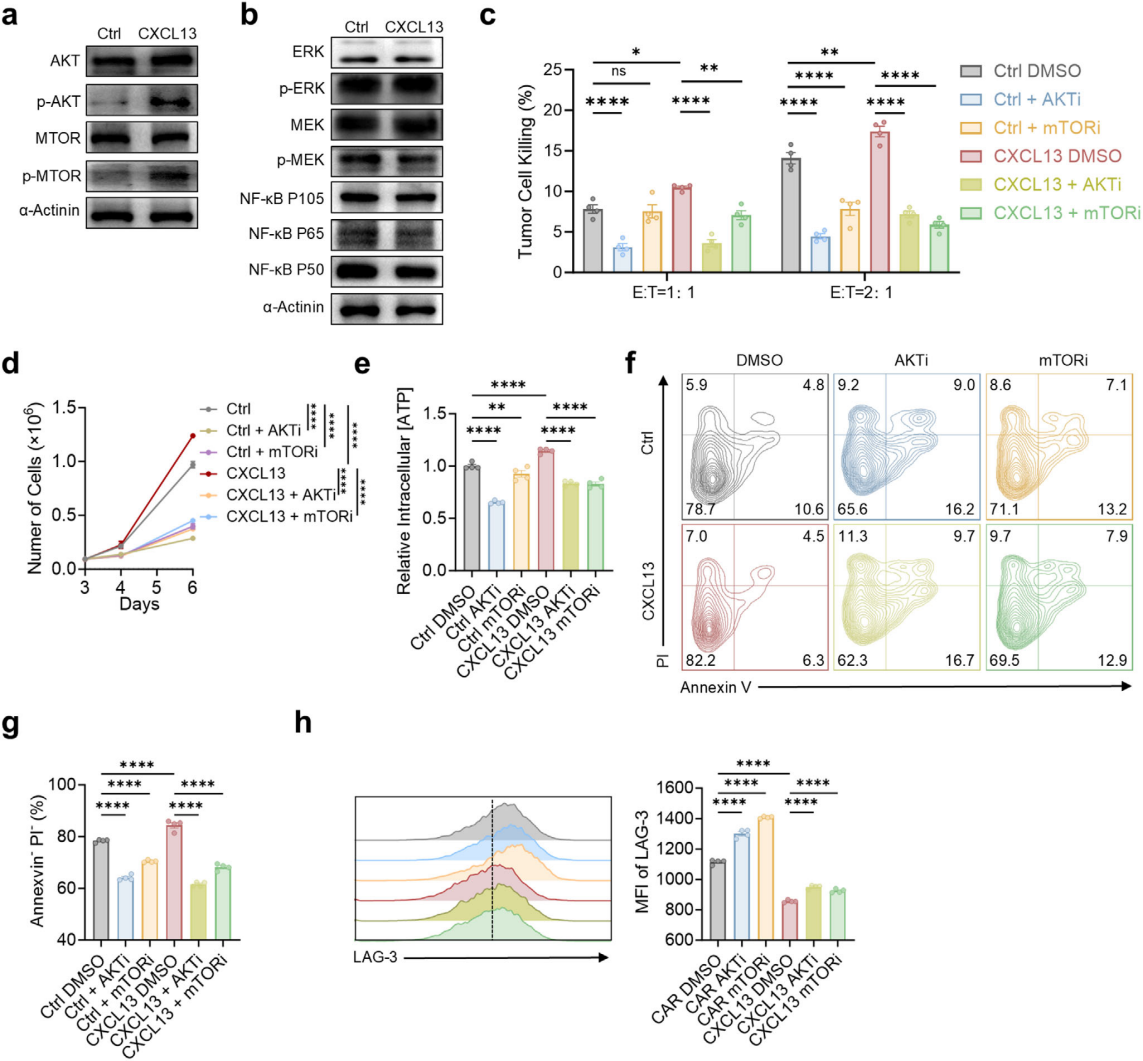
CXCL13‐CXCR5‐AKT‐mTOR signaling axis enhances cytotoxicity and proliferation while suppressing exhaustion and apoptosis. a,b). Western blot of the expression of total AKT, total mTOR, p‐AKT, p‐mTOR (a) and total ERK, total MEK, p‐ERK, p‐MEK, and NF‐κB (b). c). Cytotoxic efficacy was evaluated after an 8‐h co‐culture of CXCL13 CAR T cells or control CAR T cells with tumor cells in the presence or absence of different inhibitors. d). Cell numbers of CXCL13 CAR T cells and control CAR T cells were assessed in the presence or absence of different inhibitors. e). Bar graphs show relative intracellular ATP levels in CXCL13 CAR T cells and control CAR T cells cultured with or without different inhibitors. f,g). Apoptosis was assessed using Annexin V and PI staining followed by flow cytometry; representative flow cytometry plots were shown in (f), and bar graphs were shown in (g). h). LAG‐3 expression was analyzed by flow cytometry. *n* = 4, Mean ± SEM (c–e,g,h); ^*^ *p* < 0.05, ^**^ *p* < 0.01, ^***^ *p* < 0.001; ^****^ *p* < 0.0001; one‐way ANOVA and Tukey (c–e,g,h).

To elucidate the mechanism by which CXCL13 enhances CAR T cell function, we focused on the downstream signaling of its canonical receptor, CXCR5.^[^ ^35^ ^]^ Western blot analysis revealed that phosphorylation of AKT and mTOR was significantly increased in CXCL13 CAR T cells, while total protein levels remained unchanged (**Figure** **6**a). Other downstream CXCR5 signaling components, including ERK, MEK, and NF‐κB, showed minimal changes (Figure 6b), suggesting that AKT‐mTOR is the primary axis activated by CXCL13 in CAR T cells.

To determine the functional relevance of this pathway, we treated control and CXCL13 CAR T cells with the AKT inhibitor ipatasertib or the mTOR inhibitor rapamycin. CXCL13 CAR T cells exhibited enhanced tumor‐killing capacity relative to control cells, an effect that was substantially diminished by either inhibitor (Figure 6c). Similarly, the proliferative advantage conferred by CXCL13 was abrogated upon AKT or mTOR inhibition (Figure 6d). In parallel, CXCL13 CAR T cells showed increased ATP production, reflecting improved metabolic activity, which was also significantly reduced following pathway inhibition (Figure 6e). Notably, CXCL13 CAR T cells displayed reduced apoptosis and exhaustion compared to control cells—both of which were reversed upon AKT or mTOR blockade (Figure 6f–h).

Together, these findings establish the CXCL13‐CXCR5‐AKT/mTOR axis as a central mediator of the improved cytotoxicity, proliferation, metabolic fitness, and persistence of CXCL13 CAR T cells. While this pathway accounts for many of the observed phenotypic changes, it is possible that additional mechanisms may contribute to the full spectrum of CXCL13‐mediated effects.

### 2.7 Synergistic Effect of CXCL13 CAR T Cells with PD‐1 Blockade Therapy

**Figure 7:**
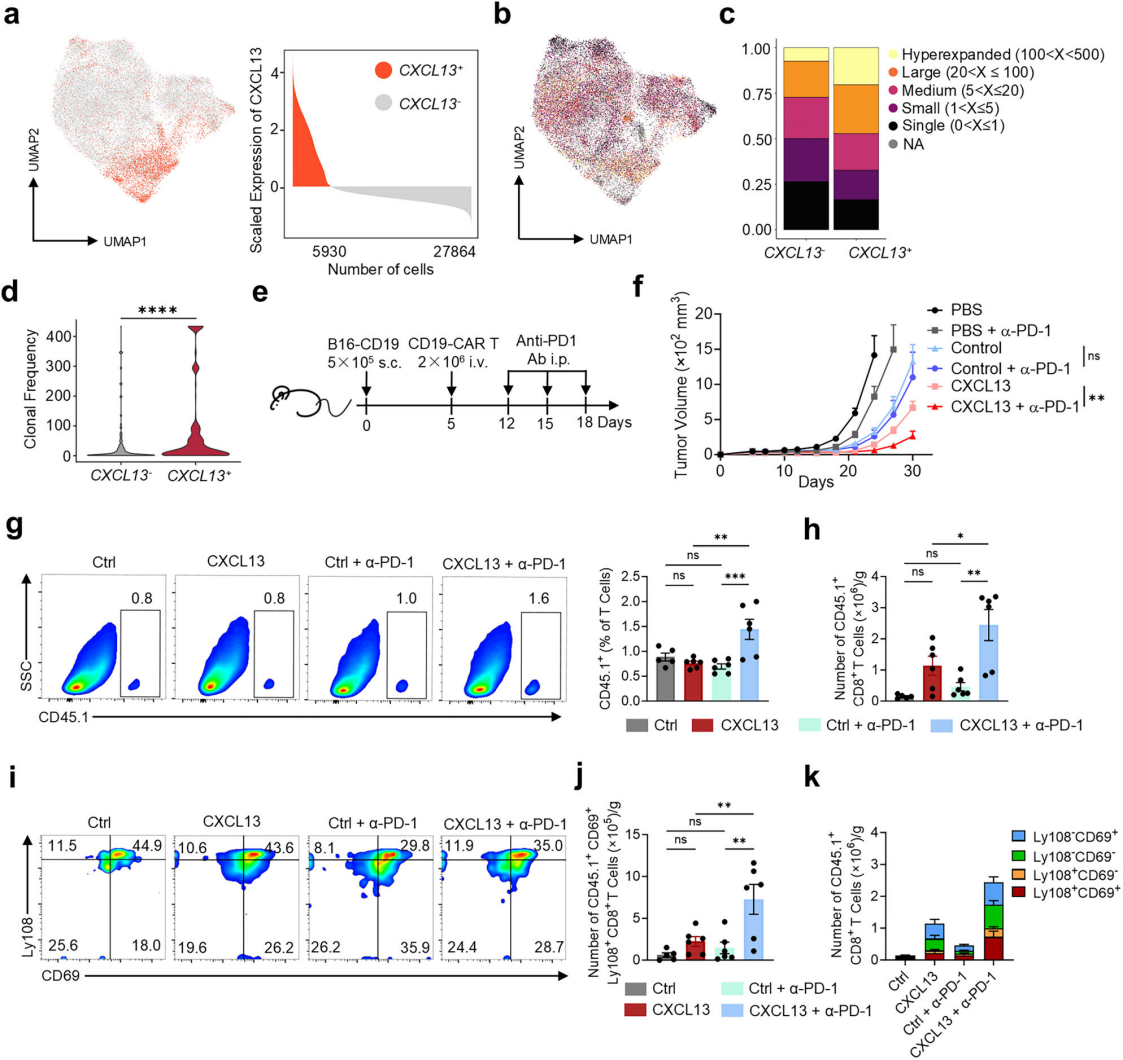
CXCL13 synergizes with anti‐PD‐1 to enhance the anti‐tumor efficacy of CAR T cells. a‐d. Single‐cell TCR analysis of CD8^+^ T cells from BCC and SCC patients accepted ICB therapy. a). UMAP plot and histogram showing *CXCL13* ^+^ and *CXCL13* ^−^ CD8^+^ T cells. b). Clone size distribution within CD8^+^ T cells. c). Clonal occupancy of *CXCL13* ^+^ and *CXCL13* ^−^ CD8^+^ T cells. d). Clonal frequency of *CXCL13* ^+^ and *CXCL13* ^−^ CD8^+^ T cells. e). Schematic of in vivo experiments. Tumor‐bearing mice were treated via intravenous (i.v.), subcutaneous (s.c.), or intraperitoneal (i.p.) injections as indicated. f). Tumor growth in subcutaneous B16‐CD19 models. Mice were treated with 2×10⁶ CAR T cells 5 days post‐tumor establishment. Groups: PBS (*n* = 6), PBS + anti‐PD‐1 (*n* = 5), control CAR T (*n* = 8), control CAR T + anti‐PD‐1 (*n* = 6), CXCL13 CAR T (*n* = 8), and CXCL13 CAR T + anti‐PD‐1 (*n* = 6). Tumor growth is expressed as the mean tumor size. Mean ± SEM. g). Flow cytometry analysis of the proportion of CD45.1^+^ cells within the CD3^+^ population in the spleen. h). Number of CD45.1^+^ CD8^+^ T cells in tumors. i). Flow cytometry plots showing Ly108 and CD69 expression on CD45.1^+^ CD8^+^ T cells in tumors. j). Number of CD45.1^+^ CD8^+^ Ly108^+^CD69^+^ T cells in tumors. k). Number of four stages of exhausted T cells in CD45.1^+^CD8^+^ T cells in tumors. *n* = 5 (control CAR T), *n* = 6 (control CAR T + anti‐PD‐1), *n* = 6 (CXCL13 CAR T), *n* = 6 (CXCL13 CAR T + anti‐PD‐1); Mean ± SEM (g,h,j,k); ^*^ *p* < 0.05, ^**^ *p* < 0.01, ^***^ *p* < 0.001; two‐tailed unpaired t‐test (d); two‐way ANOVA and Tukey (f), one‐way ANOVA and Tukey (g,h,j,k).

CAR T cell exhaustion is a critical limitation in the efficacy of solid tumor therapies, and PD‐1 blockade has shown potential efficacy to mitigate this challenge. Our integrative analysis revealed that *CXCL13* is highly expressed in patients who respond to PD‐1 blockade (Figure 1b–f), and previous studies suggest that co‐administration of CXCL13 may further enhance PD‐1 therapy efficacy.^[^ ^43^, ^44^ ^]^ CD8^+^ T cells are the most responsive to PD‐1/PD‐L1 blockade.^[^ ^45^ ^]^ To confirm that *CXCL13* ^+^ T cells respond to PD‐1 inhibition, we analyzed CD8^+^ T cells from public single‐cell TCR sequencing (scTCR‐seq) data,^[^ ^25^ ^]^ and CD8^+^ T cells were divided into two subsets based on the scaled expression of *CXCL13*: *CXCL13* ^+^ and *CXCL13* ^−^ groups (**Figure** **7**a). It was found that *CXCL13* ^+^ T cells showed greater TCR clonal expansion, which confirmed that *CXCL13* ^+^CD8^+^ T cells expand in these patients (Figure 7b–d).

To assess the potential synergy between CXCL13 CAR T cells and PD‐1 blockade, we performed both in vitro and in vivo experiments. In initial in vitro co‐culture assays, CXCL13 CAR T cells demonstrated superior tumor‐killing efficacy compared to control CAR T cells. However, adding PD‐1 blocking antibody did not enhance tumor‐killing efficacy for either control CAR T cells or CXCL13 CAR T (Figure S9a–c, Supporting Information), most likely due to the limited induction of exhaustion during short‐term in vitro co‐culture. Consequently, we conducted in vivo studies in a B16‐CD19 mouse solid tumor model, treating tumor‐bearing mice with a combination of PD‐1 blockade and CAR T cells (Figure 7e). In these experiments, control CAR T cells showed minimal response to PD‐1 blockade, while CXCL13 CAR T cells displayed a robust response, with significantly increased antitumor efficacy under PD‐1 inhibition (Figure 7f).

Following PD‐1 blockade, the proportion of CD45.1^+^ exogenous CAR T cells in the control group remained largely unchanged, whereas CXCL13 CAR T cells exhibited a marked increase in the proportion of total CD45.1^+^ CAR T cells (Figure 7g). PD‐1 blockade primarily promoted the expansion of CD45.1^+^CD8^+^ CXCL13 CAR T cells, with minimal impact on the CD4^+^ population (Figure 7h; Figure S10a,b, Supporting Information), consistent with previous findings that PD‐1 blockade predominantly affects CD8^+^ T cells. Additionally, PD‐1 blockade had little effect on endogenous CD8^+^ and CD4^+^ T cells in our tumor model (Figure S10c,d, Supporting Information), likely due to the low abundance of tumor‐specific endogenous T cells within the model.

Further analysis demonstrated that PD‐1 blockade significantly increased the proportion of early‐exhausted CD8^+^ CAR T cells within the CXCL13 CAR T group, while early exhaustion remained relatively unchanged in control CAR T cells (Figure 7i–k). This expansion of early‐exhausted cells indicates that PD‐1 blockade positively influences CXCL13 CAR T cells, enhancing their functionality and antitumor response. However, PD‐1 blockade did not significantly impact the early exhaustion state of CD45.1^+^ CD4^+^ T cells (Figure S10e,f, Supporting Information) or endogenous CD4^+^ T and CD8^+^ T cells (Figure S10g–j, Supporting Information).

In summary, CXCL13 expression enhances CAR T cell antitumor efficacy, with additional improvements in function when combined with PD‐1 blockade, underscoring CXCL13's potential as a target for optimizing CAR T therapy in solid tumors.

### 2.8 CXCL13 Enhances Antitumor‐Favoring Features in Human CAR T Cells

**Figure 8:**
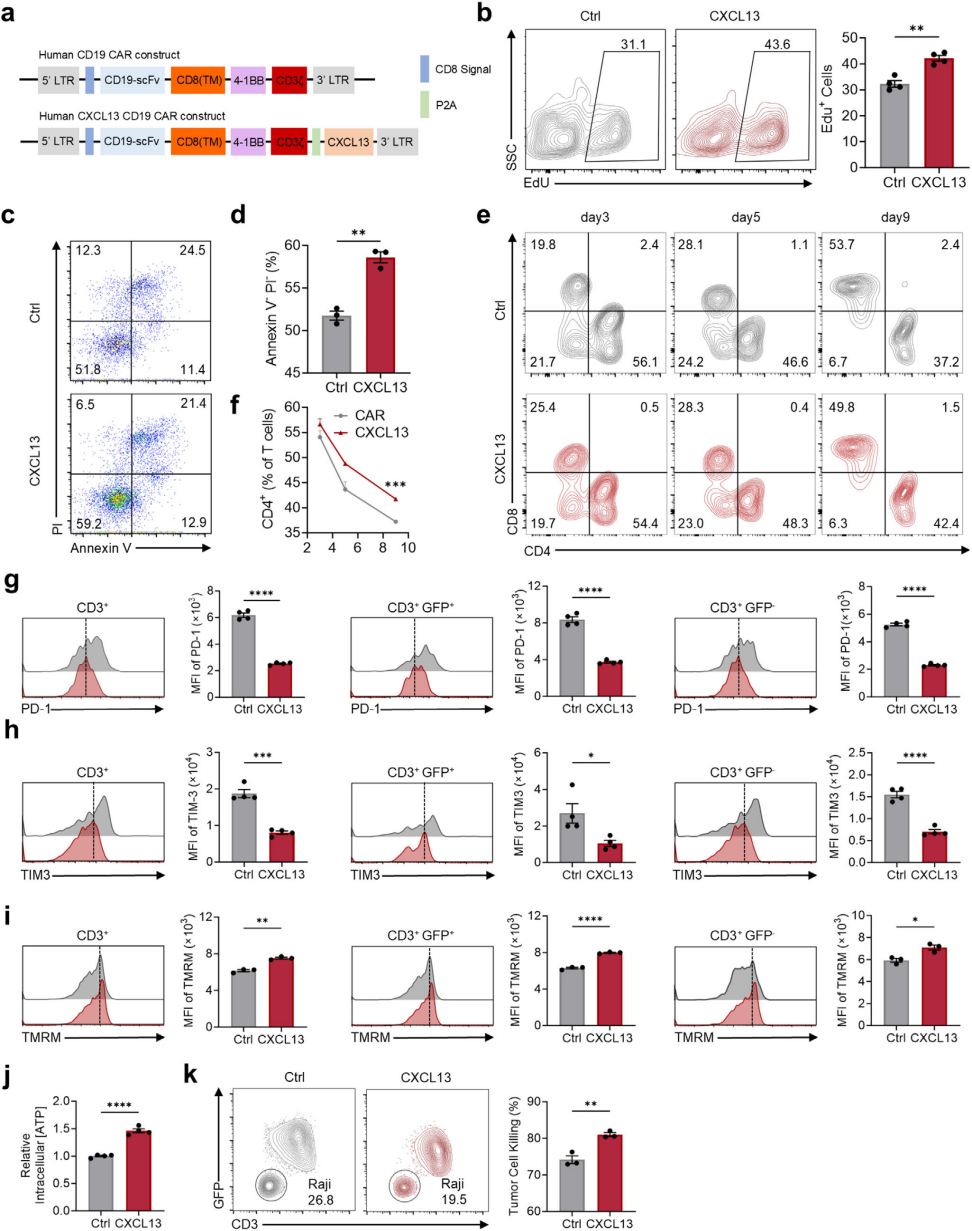
CXCL13 enhances antitumor‐favoring features in human CAR T Cells. a). Schematic representation of the structures of the human CD19 CAR and CXCL13 CD19 CAR constructs. b). Representative flow cytometry and bar graph analysis of EdU incorporation in CAR T cells. c,d). Flow cytometry plots (c) and bar graphs (d) were used to show the apoptosis levels of CAR T cells. e,f). Flow cytometry plots (e) and line graphs (f) are used to display the ratios of CD4⁺ and CD8⁺ T cells. g,h). In vitro expression levels of exhaustion‐related proteins, including PD‐1 (g) and TIM3 (h) expression. i). Flow cytometry analysis of mitochondrial membrane potential (TMRM). j). Relative intracellular ATP levels in control CAR T cells and CXCL13 CAR T cells. k). Flow cytometry analysis of tumor cell killing, with quantification of remaining Raji cells to evaluate the cytotoxic activity of control and CXCL13 CAR T cells. *n* = 3 (d,f,i,k); *n* = 4 (b,g,h,j); Mean ± SEM. ^*^ *p* < 0.05, ^**^ *p* < 0.01, ^***^ *p* < 0.001; ^****^ *p* < 0.0001; two‐tailed unpaired t‐test (b,d,f–k).

To evaluate the translational relevance of CXCL13 engineering for clinical application, we assessed the effects of CXCL13‐overexpression in human CAR T cells (**Figure** **8**a). CXCL13 significantly enhanced human CAR T cell proliferation and reduced apoptosis in vitro (Figure 8b–d), indicating improved cellular fitness. Moreover, CXCL13 CAR T cells displayed a higher proportion of CD4⁺ T cells (Figure 8e,f), a subset often associated with superior persistence and helper function in adoptive cell therapy. Functionally, CXCL13 CAR T cells demonstrated reduced expression of exhaustion markers, including PD‐1 and TIM3 (Figure 8g,h), enhanced mitochondrial health, and elevated ATP production (Figure 8i,j), which collectively contributed to their enhanced cytotoxic activity against tumor cells in vitro (Figure 8k). Notably, we observed that even the GFP⁻ (non‐transduced) population within the same cultures exhibited reduced exhaustion and enhanced mitochondrial integrity, suggesting a paracrine effect of CXCL13.

These phenotypic and functional enhancements closely mirror those observed in murine CAR T cells, suggesting a conserved mechanism of action across species. Collectively, our findings highlight the therapeutic potential of CXCL13 engineering to enhance the persistence, metabolic fitness, and antitumor efficacy of human CAR T cells, supporting its further evaluation in preclinical models and eventual clinical translation.

## 3 Discussion

Multiple strategies have been developed to enhance T cell functionality in tumor immunotherapy, including ICB and T cell engineering.^[^ ^7^, ^46^ ^]^ Despite these advances, achieving robust and durable immunotherapeutic efficacy remains a significant challenge, particularly in the context of solid tumors.^[^ ^4^, ^6^, ^8^ ^]^ Combining different therapeutic strategies represents a promising approach. Here, we demonstrate that CXCL13‐overexpression not only significantly enhances the antitumor activity of CAR T cells but also sensitizes them to PD‐1 blockade, resulting in a synergistic therapeutic effect.

The limited efficacy of CAR T therapy in solid tumors is primarily due to T cell exhaustion, insufficient persistence, and inadequate tumor infiltration.^[^ ^7^ ^]^ In the TME, CAR T cells are subjected to prolonged antigen stimulation, leading to terminal differentiation and exhaustion, which compromises their cytotoxic capacity.^[^ ^47^ ^]^ While the role of CXCL13 in T cell recruitment is well‐documented,^[^ ^35^ ^]^ its effects on T cell phenotypes are less understood. Our findings reveal that CXCL13‐overexpressing CAR T cells exhibit reduced exhaustion and an enhanced central memory (TCM) phenotype both in vitro and in vivo. This phenotype, characterized by long‐lived and proliferative potential, is critical for maintaining CAR T cell activity over extended periods.^[^ ^48^, ^49^ ^]^ Furthermore, CXCL13 CAR T cells display improved mitochondrial function, supporting their metabolic fitness and resistance to exhaustion in the TME.^[^ ^50^, ^51^ ^]^ Interestingly, while previous studies suggest that CXCL13 can promote tumor cell proliferation and survival in vitro,^[^ ^52^ ^]^ our CXCL13 CAR T cells demonstrate reduced apoptosis and increased proliferative potential. These properties collectively contribute to their superior persistence and heightened cytotoxicity, effectively addressing several critical limitations faced by conventional CAR T therapies in solid tumors.

T cell activation naturally upregulates inhibitory co‐stimulatory molecules such as PD‐1, which maintain immune homeostasis by antagonizing TCR and γc‐mediated signaling.^[^ ^53^ ^]^ However, in the TME, persistent PD‐1 expression in T cells leads to exhaustion and dysfunction.^[^ ^54^ ^]^ PD‐1 blockade has been shown to reinvigorate exhausted T cells and promote T cell proliferation, thereby enhancing tumor control.^[^ ^5^ ^]^ Nonetheless, not all patients benefit from ICB therapy, and even in responders, only a small subset of CD8^+^ T cells, often characterized by CXCR5 or CXCL13 expression, exhibit robust proliferative responses.^[^ ^16^, ^55^ ^]^ The precise features of these ICB‐responsive T cells remain poorly understood.

Preclinical and clinical studies have shown limited synergy between PD‐1 blockade and ACT in solid tumors. Transferred T cells in animal models often fail to respond to PD‐1 blockade^[^ ^56^ ^]^ and clinical trials demonstrated that PD‐1 inhibition has minimal impact on CAR T cell growth or persistence in solid tumor settings, such as neuroblastoma,^[^ ^13^ ^]^ glioblastoma,^[^ ^14^ ^]^ and mesothelin‐targeting CAR T therapies.^[^ ^15^ ^]^ These findings highlight the need to identify and leverage the unique features of PD‐1‐responsive T cell populations.

ScRNA‐seq has enabled the identification of rare T cell populations with superior anti‐tumor efficacy in the TME, including *CXCL13* ^+^ T cells, which are increasingly recognized for their responsiveness to ICB.^[^ ^17^, ^21^ ^]^ CXCL13, a chemokine primarily interacting with CXCR5, is secreted by T cells and acts on B cells and T cells within the TME,^[^ ^35^, ^57^ ^]^ which is consistent with our findings in scRNA‐seq analysis. While CXCL13 is known to recruit immune cells and facilitate the formation of tertiary lymphoid structures (TLS),^[^ ^35^ ^]^ we observed increased B cell infiltration in tumors treated with CXCL13‐overexpressing CAR T cells, raising the possibility that CXCL13 may enhance PD‐1 blockade responsiveness through B cell‐dependent mechanisms, such as facilitating tertiary lymphoid structure (TLS) formation. However, this possibility is challenged by the observation that CXCL13 CD19 CAR T cells—despite actively depleting B cells—still exhibited superior antitumor efficacy. These findings suggest that the therapeutic benefits of CXCL13 are not solely dependent on B cell recruitment or TLS‐related pathways. Instead, CXCL13 may act through T cell‐intrinsic mechanisms or modulate other aspects of the tumor microenvironment (TME) that augment responsiveness to PD‐1 blockade. Supporting this, we did not detect significant changes in the frequencies of endogenous CD4⁺ T cells, CD8⁺ T cells, or F4/80⁺ macrophages in tumors treated with CXCL13 CAR T cells. Together, these results indicate that the enhanced efficacy of CXCL13 CAR T cells likely arises from a combination of direct functional enhancement and TME remodeling, independent of B cell involvement in the CD19‐targeting context. Further studies in non‐B cell‐depleting models are warranted to fully elucidate the role of B cells and other immune subsets in CXCL13‐mediated antitumor immunity.

It has been reported that tumor‐draining lymph node (TDLN)‐derived tumor‐specific memory T cells are the primary responders to PD‐1/PD‐L1 ICB.^[^ ^45^ ^]^ Furthermore, PD‐1/PD‐L1 blockade preferentially expands precursor‐exhausted T cells rather than terminally exhausted T cells.^[^ ^22^ ^]^ Our results indicate that CXCL13‐overexpression enhances the central memory phenotype of CAR T cells and reduces exhaustion, enabling them to respond more effectively to PD‐1 blockade. CXCL13 CAR T cells also show an expansion of CD8^+^ T cells with an early exhausted phenotype, which likely supports their sustained antitumor activity. The immunosuppressive TME also limits the efficacy of PD‐1 blockade by restricting the infiltration and activation of tumor‐reactive T cells.^[^ ^5^ ^]^ Our data suggest that CXCL13 CAR T cells overcome these barriers through enhanced migration and infiltration into the TME. Furthermore, in human CAR T cells, CXCL13‐overexpression improved proliferation, cytotoxicity, mitochondrial function, while reducing exhaustion and apoptosis—highlighting its translational potential for improving CAR T therapy in solid tumors. These findings align with previous studies demonstrating that intraperitoneal injection of CXCL13 enhances the efficacy of PD‐1 therapy,^[^ ^44^ ^]^ further supporting the synergistic potential of combining CXCL13 CAR T cells with PD‐1 blockade. However, CAR T cells did not respond to PD‐1 blockade during short‐term (14‐hour) in vitro co‐culture with tumor cells, whereas in vivo, tumor‐infiltrating CAR T cells exhibited enhanced proliferation following anti‐PD‐1 treatment. This discrepancy likely reflects insufficient exhaustion induction in vitro, where brief antigen exposure fails to recapitulate the chronic stimulation and immunosuppressive cues present in the tumor microenvironment. In contrast, sustained antigen exposure in vivo promotes functional exhaustion, rendering CAR T cells more responsive to PD‐1 blockade. These findings highlight the critical role of the in vivo tumor microenvironment in modeling T cell exhaustion and evaluating the efficacy of immune checkpoint therapies.

In summary, our study highlights the multifaceted benefits of CXCL13‐overexpression in CAR T cells, including reduced exhaustion, enhanced memory phenotypes, improved metabolic fitness, and superior migratory capacity—effects largely mediated through the AKT‐mTOR signaling axis. These features collectively enable more potent and durable antitumor responses in solid tumors. Moreover, CXCL13 CAR T cells exhibited synergistic activity when combined with PD‐1 blockade, further enhancing therapeutic efficacy. Importantly, human CXCL13 CAR T cells recapitulated the key functional advantages observed in murine models, including enhanced proliferation, reduced exhaustion, and improved cytotoxicity, supporting the translational relevance of this strategy. However, due to the lack of suitable immunocompetent or fully humanized mouse models, we were unable to assess the in vivo efficacy of human CXCL13 CAR T cells. Future studies should prioritize the development of appropriate preclinical systems to evaluate therapeutic performance and safety, which will be critical for advancing CXCL13 CAR T cell therapy toward clinical application.

## 4 Experimental Section

### Single‐Cell RNA‐seq Data Analysis

Single‐cell RNA sequencing (scRNA‐seq) data from patients receiving immune checkpoint inhibitors and CAR T cell treatments were collected.^[^ ^19^, ^20^, ^21^, ^22^, ^23^, ^24^, ^25^, ^32^, ^33^, ^34^, ^42^ ^]^ First, expression levels were normalized using the “NormalizeData” function in the R package Seurat (5.1.0).^[^ ^58^ ^]^ After identifying highly variable features and scaling the data, principal component analysis (PCA) was used to reduce dimension. The K‐nearest neighbor (KNN) graph was constructed based on the Euclidean distance using the selected principal components, following optimization of intercellular distance weights using Jaccard similarity. The Louvain algorithm was then applied to cluster the data. The UMAP (Uniform Manifold Approximation and Projection) was used to show the results. Cell subpopulations were further annotated based on the expression profiles of characteristic marker genes. The “DotPlot” function to visualize CXCL13 expression levels in responders versus non‐responders was utilized and performed differential gene expression analysis using the “FindMarkers” function in Seurat. Different genes were identified under the following criteria: *p*.adjust < 0.05; log‐transformed fold change (Log_2_FC) ≥ 0.25 or ≤ ‐0.25; and percentage expression difference (i.e., the percentage of cells expressing the gene in responders minus that in non‐responders) ≥ 0.025 or ≤ ‐0.025. Gene expression density was calculated using the R package Nebulosa.^[^ ^59^ ^]^

### Kaplan‐Meier Survival Curve Analysis

R packages survminer (0.4.9) and survival (3.6‐4) were used to calculate Kaplan‐Meier survival curves for bladder cancer and melanoma immunotherapy cohorts. The website Kaplan‐Meier Plotter^[^ ^60^ ^]^ was applied to compute survival curves for READ, UCEC, STAD, OV, HNSC, BRCA, COAD, CESC, and BLCA.

### Bulk RNA seq

Control CAR T cells and CXCL13 CAR T cells were cultured in vitro until day 5, and RNA was extracted using AG RNAex Pro Reagent (AG, Cat # AG21102). RNA quality was assessed by Biomarker Technologies, followed by library construction and sequencing on the Illumina NovaSeq6000 platform. Raw fastq data were aligned to the GRCm38 genome using hisat2, and quantified with featureCounts to generate counts data, which were subsequently normalized as FPKM (Fragments Per Kilobase of transcript per Million mapped fragments). Differential gene expression analysis between the two groups was performed using the R package DESeq2 (1.44.0).^[^ ^61^ ^]^ Different genes were identified under the following criteria: *p*.adjust < 0.05; fold change ≥ 1.5 or ≤ ‐1.5. The volcano plot was performed using the R package ggplot2 (3.5.1). The R package pheatmap (1.0.12) was used to plot the heatmap (scale = raw).

### Functional Enrichment Analysis

GSEA (Gene Set Enrichment Analysis) was performed using the R package ClusterProfiler (4.12)^[^ ^62^ ^]^ based on the GO and KEGG databases. R package GseaVis (0.1.0) was used to show GSEA plots.

### Cellular Communication

CellChat (1.6.1)^[^ ^63^ ^]^ was used to evaluate the CXCL13/CXCR5 axis among all subtypes, and intercellular connections between T cells and other cell subtypes.

### Mouse and Cell Lines

The Laboratory Animal Center at Southern Medical University supplied C57BL/6 mice, and Professor Bing Sun generously provided CD45.1 (B6.SJL‐Ptprca Pepcb/BoyJ) mice. Cell lines, including B16‐CD19, 293T, MC38, and MC38‐CD19, B16‐CLDN18.2, MB49‐CD19 were maintained in Dulbecco's Modified Eagle Medium (DMEM, Biological Industries) containing 10% heat‐inactivated fetal bovine serum (FBS, Gibco) and 100 U mL^−1^ penicillin‐streptomycin (Gibco). Raji cells were cultured in RPMI 1640 Medium (Gibco) containing 10% heat‐inactivated fetal bovine serum (FBS, Gibco) and 100 U/ml penicillin‐streptomycin (Gibco). All animal experiments were carried out in compliance with approved protocols by the Institutional Animal Care and Use Committee of Southern Medical University (Approval No. SMUL202406037).

### Mouse T Cell Culture

CD3^+^ T cells were isolated from splenocytes of C57BL/6J or CD45.1 mice using the MojoSort Mouse CD3 T Cell Isolation Kit (Biolegend, Cat # 480 031). T cells were activated by culturing on plates coated with anti‐CD3 (Bioxcell, 17A2, 5 µg mL^−1^) in the presence of soluble anti‐CD28 (Bioxcell, 37.51, 1 µg mL^−1^) and IL‐2 (Novoprotein, C013, 100 U mL^−1^) to induce differentiation. Cells were maintained in RPMI 1640 medium supplemented with 10% fetal bovine serum (FBS), 100 U mL^−1^ penicillin‐streptomycin (Gibco, Cat # 15 140 122), 1×GlutaMAX (Gibco, Cat # 35 050 061), and 1×2‐Mercaptoethanol (Gibco, Cat # 21 985 023) at 37 °C and 5% CO₂. After three days, T cells were gently detached from plates, centrifuged to remove the supernatant, and further cultured in fresh medium containing IL‐2 (Novoprotein, C013, 100 U mL^−1^).

To verify the role of the AKT‐mTOR pathway in CXCL13 CAR T cells, cells were treated with either the AKT inhibitor Ipatasertib (10 µm; MCE, Cat # GDC‐0068) or the mTOR inhibitor rapamycin (20 nm; Enzo, Cat # 51031‐RAP‐25), and subsequently subjected to downstream experiments.

### Human T Cell Culture

Human CD3^+^ T cell was isolated from PBMCs of healthy volunteer donors using MojoSort Human CD3 Selection Kit (Biolegend, Cat # 480 134). T cells were activated by culturing on plates coated with anti‐CD3 (Biolegend, Cat # 317 326, 5 µg mL^−1^) in the presence of soluble anti‐CD28 (Biolegend, Cat # 377 304, 2 µg mL^−1^). Human T cells were cultured in OptiVitro T Cell Medium SF (Excell Bio, TE000‐N022) supplemented with IL‐2 (Novoprotein, C013, 100 U mL^−1^) at 37 °C and 5% CO_2_. The study was performed according to the guidelines of the Medical Ethics Committee of NanFang Hospital of Southern Medical University (Approval No. NFEC‐2025‐131).

### Plasmids

For mouse T cells, the second‐generation CAR contains a single‐chain variable fragment (scFv) targeting mouse CD19, along with hinge, transmembrane, and intracellular domain of CD28 and CD3ζ intracellular signaling domain which was constructed into the Retroviral vector MigR1 (Addgene, #27 490). For anti‐Claudin 18.2 CAR, the scFv consisted of a variable heavy chain (VH) and a variable light chain linked by GGGGS×3.

For human T cells, second‐generation CAR contains an scFv targeting human CD19, along with CD8 hinge and transmembrane domains, 4‐1BB, and CD3ζ intracellular signaling domains, and was constructed into lentiviral vector pUltra (Addgene, #24 129).

For CXCL13‐overexpression, both human and mouse CXCL13 were designed and modified to include a CXCL13 signal peptide and a self‐cleaving 2A peptide (P2A).

### Viral Production and Transduction

293T cells were seeded on 6‐well plates and co‐transfected with retroviral vector plasmid, packaging plamid pCL‐ECO (Addgene, #12 371) using Lipo3000 (Thermo Fisher, Cat # L3000075) or co‐transfected with lentiviral vector plasmid, packaging plamid pMD2.G (Addgene, #12 259) and psPAX2 (Addgene, #12 260,) using X‐tremeGENE HP DNA Transfection Reagent (Roche, Cat # 0 636 623 6001). Viral supernatants were harvested 48 h post‐transfection and stored at −80 °C for future use.

For both human and mouse CAR T, the transduction was performed after 40 h of incubation (MOI = 5). CD3^+^ T cells were then cultured with viral particles and polybrene (10 µg mL^−1^, Sigma–Aldrich. Cat # TR‐1003), and centrifuged at 2000 rpm for 120 min in a cell culture plate, followed by incubation for 24 h. In CAR T cell studies, ≈40%–80% of T cells exhibited GFP positivity post‐viral transduction.

### Tumor Inoculation and Therapy

C57BL/6 mice were subcutaneously injected with tumor cells. On day 3 post‐tumor inoculation, mice received cyclophosphamide (CTX, Sigma, Cat # PHR1404‐1G) to deplete peripheral lymphocytes. On day 5, mice were divided into groups and administered either PBS, control CAR T cells, or CXCL13 CAR T cells via tail vein injection. Tumor growth and mouse survival were monitored every 3 days. For experiments involving CAR T cells combined with ICB, tumor‐bearing mice received CAR T cell treatment as described above. One week following adoptive cell transfer (ACT), mice received intraperitoneal injections of anti‐PD‐1 antibody at a total dose of 200 µg per mouse, 100 µg on day 7, 50 µg on day 10, and 50 µg on day 13 post‐ACT.

### Isolation of Animal Tissues

Single‐cell suspensions were obtained by cutting tumors, draining lymph nodes, and spleens into small pieces and passing them through a 70‐µm cell strainer. The suspensions were prepared for analysis with FACS buffer. For blood and spleen samples, before FACS staining, red blood cells in suspensions were destroyed using red blood cell lysis solution (Solarbio, Cat # R1010‐500 mL). T cell counts were determined using Precision Count Beads (Biolegend, Cat # 42 490).

### Flow Cytometry

After centrifuging the supernatant and washing the cells with FACS buffer, the cells were prepared for cell surface staining. Surface staining was then performed by incubating cells in a pre‐diluted antibody cocktail, either in 96‐well V‐bottom plates or 1.5 mL Eppendorf tubes, on ice and protected from light for 30 min. Staining was stopped by adding FACS buffer, followed by centrifugation to remove the supernatant, and cells were washed twice in FACS buffer before resuspension. The following antibodies were used for staining: anti‐mouse CD45.1, BV450 (E‐AB‐F1184UQ), Elabscience, Cat # AF19855; anti‐mouse CD45.1, PE (A20), Biolegend, Cat # 110 708; anti‐mouse CD3ε, PE/Cyanine7 (145‐2C11), Biolegend, Cat # 100 320; anti‐mouse PD‐1, PE/Cyanine7 (RMP1‐30), Biolegend, Cat # 109 110; anti‐mouse CD4, APC/Cy7 (E‐AB‐F1353UJ), Elabscience, Cat # AF18028; anti‐mouse CD4, PerCP/Cyanine5.5 (RM4‐5), Biolegend, Cat # 100 540; anti‐mouse CD4, PE/Cyanine7 (RM4‐5), Biolegend, Cat # 100 527; anti‐mouse CD4, Brilliant Violet 785 (RM4‐5), Biolegend, Cat # 100 552; anti‐mouse CD8a, PE (53‐6.7), Biolegend, Cat # 100 708; anti‐mouse CD8a, APC (53‐6.7), Biolegend, Cat # 100 712; anti‐mouse CD8a, PE/Cyanine7 (53‐6.7), Biolegend, Cat # 100 721; anti‐mouse CD45, PerCP/Cyanine5.5 (30‐F11), Biolegend, Cat # 103 132; anti‐mouse Ly108, PE (330‐AJ), Biolegend, Cat # 134 605; anti‐mouse LAG‐3, PerCP/Cyanine5.5 (C9B7W), Biolegend, Cat # 125 211; anti‐mouse LAG‐3, APC (C9B7W), Biolegend, Cat # 125 210; anti‐mouse CD69, Brilliant Violet 605 (H1.2F3), Biolegend, Cat # 104 530; anti‐mouse/human CD44, Brilliant Violet 510 (IM7), Biolegend, Cat # 103 044; anti‐mouse CD62L, PerCP/Cyanine5.5 (MEL‐14), Biolegend, Cat # 104 432; anti‐mouse CTLA‐4, APC (UC10‐4B9), Biolegend, Cat # 106 309; anti‐human CD3, APC (OKT3), Biolegend, Cat # 317 318; anti‐human CD4, PerCP/Cyanine5.5 (RPA‐T4), Biolegend, Cat # 300 530; anti‐human CD8, APC/Cyanine7 (SK1), Biolegend, Cat # 344 713; anti‐human CD279(PD‐1), PE (A17188A), Biolegend, Cat # 379 209; anti‐human CD366(Tim3), PE/CY7 (F38‐2E2), Biolegend, Cat # 345 014. Fixable Viability Dye eFluor 660 (Thermo Fisher, Cat # 65‐0864‐18) and Fixable Viability Dye eFluor 780 (Thermo Fisher, Cat # 65‐0865‐18) were used to label dead cells following the manufacturer's instructions. As directed by the manufacturer, the APC Annexin V Apoptosis Detection Kit with PI (Biolegend, Cat # 640 932) was utilized to identify necrotic and apoptotic cells. TMRM (tetramethylrhodamine, methyl ester) (Macklin, Cat # T868125) was used to stain mitochondrial membrane potential.

The BD LSRFortessa X‐20, BD Canto II, or CytoFLEX was used to examine samples, with data analysis performed using FlowJo software.

### Immunofluorescence

Tumor tissues were excised from mice bearing MB49‐CD19 tumors. The excised tumor tissues were embedded in optimal cutting temperature compound (OCT) and then frozen at −80 °C. Frozen tissue sections were prepared using a cryostat (Thermo, CRYOSTAR NX50), dried at 37 °C for 10–20 min, and fixed in methanol for 30 min. After PBS washes, antigen retrieval was performed under optimized conditions, avoiding tissue drying. Sections were cooled to room temperature and washed again with PBS. Tissue boundaries were marked with a hydrophobic barrier pen. Endogenous peroxidase activity was blocked using 3% H₂O₂ for 25 min in the dark. Non‐specific binding was blocked with 10% rabbit serum (for goat antibodies) or 3% BSA for 30 min at room temperature. Primary antibodies: F4/80 (ServiceBio, Cat # GB113373‐100), CD3 (ServiceBio, Cat # GB12014‐100), and CD45.1 (Proteintech, Cat # 84325‐2‐RR) were applied sequentially with overnight incubation at 4 °C in a humidified dark chamber. After PBS washes, HRP‐conjugated secondary antibodies were applied for 50 min at room temperature, followed by TSA development (10 min, dark) and TBST washes. Antibody stripping was performed twice with elution buffer (5 min at RT, then 30 min at 37 °C), followed by TBST washes. This staining cycle was repeated for each target. Finally, nuclei were counterstained with DAPI (10 min, dark).

### Western Blot

Cells were washed with PBS twice and then lysed in 1×lysis buffer (NCM, Cat # WB3100) with protease cocktail (Solarbio, Cat # P1260). Cell lysates were resolved by SDS‐polyacrylamide gel electrophoresis and then analyzed by Western blotting with the following antibodies: MEK1/2 (Cell Signaling Technology, Cat # 4694S), p‐MEK1/2 (Cell Signaling Technology, Cat # 9154S), ERK1/2 (Biolegend, 686 902), p‐ERK1/2 (Biolegend, 369 502), Akt (Cell Signaling Technology, Cat # 4691T), p‐Akt (Biolegend, Cat # 649 001), NF‐κB1, p105, p50 (Proteintech, Cat # 14220‐1‐AP), NF‐κB, p65 (Proteintech, 10745‐1‐AP), mTOR (Biolegend, Cat # 659 202), p‐mTOR (Cell Signaling Technology, Cat # 5536T), α‐Actinin (ServiceBio, Cat # GB11230‐100), Goat Anti‐Rabbit IgG(H&L) (YEASEN, Cat # 33101ES60), Goat anti‐Mouse IgG (H&L) (ZEN‐BIOSCIENCE, Cat # 511 103), Goat anti‐Rat IgG (H&L) (Proteintech, Cat # SA00001‐15).

### EdU Assay

To evaluate cell proliferation, cultured cells were incubated with 10 µm EdU (Elabscience, Cat # E‐CK‐A373) for 2 h at 37 °C. Following incubation, cells were harvested and washed twice with PBS containing 1% BSA. Cells were then fixed in 4% paraformaldehyde for 15 min at room temperature in the dark and permeabilized with PBS containing 0.1% Triton X‐100 for 20 min. Click reaction was carried out by incubating cells with 500 µL of freshly prepared Click reaction solution containing Elab Fluor 647 Azide, CuSO₄, Click Additive, and Reaction Buffer I, according to the manufacturer's instructions. After 30 min of incubation at room temperature in the dark, cells were washed and resuspended in PBS containing 1% BSA. EdU‐positive cells were analyzed by flow cytometry, and data were processed using FlowJo software.

### Proliferation Assay

Following the initial 3 days of stimulation, T cells were collected, and stained using CellTrace Far Red proliferation kit (Invitrogen, Cat # C34572) as per the manufacturer instructions. The Far Red‐labeled T cells were then cultured with IL‐2 (100 U/mL). Samples were collected and analyzed by flow cytometry one and two days after staining.

### T Cell Transwell Assay

MC38‐CD19 cells (1 × 10⁵) were seeded in a 24‐well plate and incubated overnight. The following day, CAR T cells (5 × 10⁵) in T cell culture medium (TCM) were added to the transwell chamber (Corning, Cat # 3421) placed above the tumor cells, ensuring that the medium level covered the inserts. After co‐culturing for 14 h, the chambers were removed, and the proportion of CAR T cells in the 24‐well plate was assessed.

### In Vitro Cytotoxicity Assay

For mouse CAR T cells, the mixture of MC38‐CD19 (5 × 10^4^) and MC38 (5 × 10^4^) cells was seeded into a 24‐well plate. The following day, CAR T cells were added at designated effector‐to‐target (E: T) ratios according to experimental group allocations, with or without the addition of anti‐PD‐1 (20 µg mL^−1^). After co‐culture, remaining cells were collected, and CD3 antibodies and Fixable Viability Dye eFluor 780 staining were performed to determine the number of surviving cells and assess cytotoxic efficacy.

For human CAR T cells, Raji cells were seeded in 24‐well plates at a density of 1 × 10^5^ cells well^−1^. 5 × 10^4^ CAR T cells per well were added to Raji‐containing wells at an effector‐to‐target (E: T) ratio of 0.5:1. Wells containing only Raji cells served as controls. After 14 h of co‐culture, cell suspensions were collected, stained with Fixable Viability Dye eFluor 780 and CD3 antibodies, and mixed with Precision Count Beads. Flow cytometry was used to detect residual Raji cells, and cytotoxicity was calculated based on Raji‐only control wells.

### Real‐Time PCR

The RNAex Pro RNA Reagent (AG, Cat # AG21102) was used to extract total RNA from T cells. The Hifair III 1st Strand cDNA Synthesis SuperMix (YEASEN, Cat # 11137ES10) was then used for cDNA synthesis. Quantitative PCR was carried out on a QuantStudio 3 Real‐Time PCR System (Thermo Fisher) using primers from Sangon and ChamQ Universal SYBR qPCR Master Mix (Vazyme, Cat # Q711‐02). *Gapdh* served as the housekeeping gene to normalize mRNA expression levels. *Gapdh* F: GACAACTTTGGCATTGTGG, *Gapdh* R: ATGCAGGGATGATGTTCTG; *Cxcl13* F: CTCTCCAGGCCACGGTATTC, *Cxcl13* R: TTTGGCACGAGGATTCACAC.

### Intracellular ATP Assay

T cells were gently detached from the culture plate, collected by centrifugation, and the cell pellet was carefully resuspended. The experiment was performed according to the instructions of the ATP Assay Kit (Beyotime, Cat # S0026), and was measured using a microplate plate reader (TECAN, Infinite M200) after a few seconds.

### Data Analysis

Statistical analysis employed Student's t‐test, one‐way ANOVA, and two‐way ANOVA using Prism 10.1.2. A *p*‐value less than 0.05 was considered statistically significant.

### Declaration of Generative AI and AI‐Assisted Technologies in the Writing Process

During the preparation of this work, the authors used ChatGPT in order to improve the readability and language of the manuscript. After using this tool, authors reviewed and edited the content as needed and took full responsibility for the content of the published article.

## Conflict of Interest

The authors declare no conflict of interest.

## Author Contributions

Y.Z, W.Z., Y.Z., and H.L. contributed equally to this work. X.Z., E.B., and Y.H. initiated the study, designed the experiments, and wrote the paper; Y.Z. performed all the bioinformatic analysis; Y.Z., W.Z., Y.Z., and H.L. performed most of the experiments and statistical analyses; Y.S., Z.G., X.L., Z.L., K.W., Y.W., J.R., and R.X. helped with the experiments, J.L. provided critical suggestions.

## Supporting information

Supporting Information

Supplementary Table

## Data Availability

The bulk RNA‐seq data from this study can be found at GSE283222. All other data are provided in the article and/or supplement. Previously published scRNA‐seq datasets of ICB‐treated patients reanalyzed here are available under accession codes GSE149652 (BLCA data^[^ ^19^ ^]^), GSE195832 (HNSCC data^[^ ^20^ ^]^), GSE123813 (BCC and SCC data^[^ ^25^ ^]^), GSE169246 (TNBC data^[^ ^21^ ^]^), and GSE179994 (NSCLC data^[^ ^22^ ^]^). The PRAD data^[^ ^23^ ^]^ were downloaded from https://data.mendeley.com/datasets/5nnw8xrh5m/1. The RCC data^[^ ^24^ ^]^ were downloaded from https://singlecell.broadinstitute.org/single_cell/study/SCP1288/tumor‐and‐immune‐reprogramming‐during‐immunotherapy‐in‐advanced‐renal‐cell‐carcinoma#study‐summary. Previously published scRNA‐seq datasets of CAR T treated patients reanalyzed here are available under accession codes: GSE253352 (GPC3‐positive solid tumors data^[^ ^33^ ^]^), GSE186802 (gliomas data^[^ ^32^ ^]^), GSE262072 (ALL data^[^ ^34^ ^]^), and GSE223071 (neuroblastoma data^[^ ^42^ ^]^). Previously published bulk RNA‐seq datasets of ICB are available: PRJEB23709 (Melanoma cohort1 and cohort2^[^ ^27^ ^]^), GSE93157 (HNSC^[^ ^30^ ^]^), PRJEB25780 (STAD^[^ ^28^ ^]^), R package IMvigor210CoreBiologies (BLCA^[^ ^29^ ^]^).
