# Supplementary material for: CXCL13 Expression Promotes CAR T Cell Antitumor Activity and Potentiates Response to PD‐1 Blockade
Source: Adv Sci (Weinh). 2025 Jun 10;12(33):e08095. doi: 10.1002/advs.202508095 (PMC12412523; doi:10.1002/advs.202508095)
Supplement: Supplementary file 1 — Supporting Information [file ADVS-12-e08095-s002.pdf]

ADVANCED SCIENCE
Open Access

Supporting Information

for *Adv. Sci.*, DOI 10.1002/advs.202508095

CXCL13 Expression Promotes CAR T Cell Antitumor Activity and Potentiates Response to PD-1 Blockade

Yang Zhou, Wenli Zhao, Yihan Zhu, Hongyan Liu, Yicheng Sun, Zhengrong Gong, Xuanyi Li, Ziying Liu, Kang Wen, Yicheng Wang, Jie Ren, Ruipei Xiao, Ling Jiang, Yanfeng Hu, Enguang Bi* and Xiaoyong Zhang**

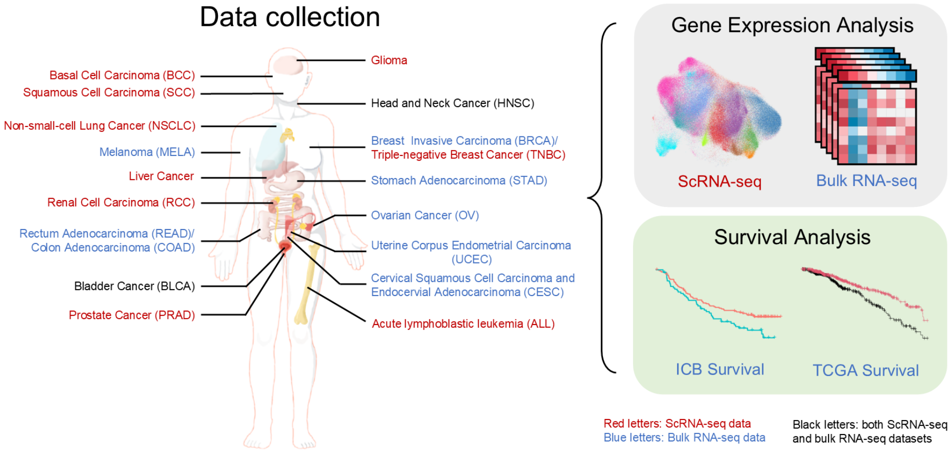

Fig. S1. Overview of data analysis. Schematic representation of collection of scRNA-seq and bulk RNA-seq data from various cancer types. Red text indicates scRNA-seq datasets, blue text represents bulk RNA-seq datasets, and black text denotes datasets available in both formats. Gene expression analysis and survival assays were conducted using these datasets.

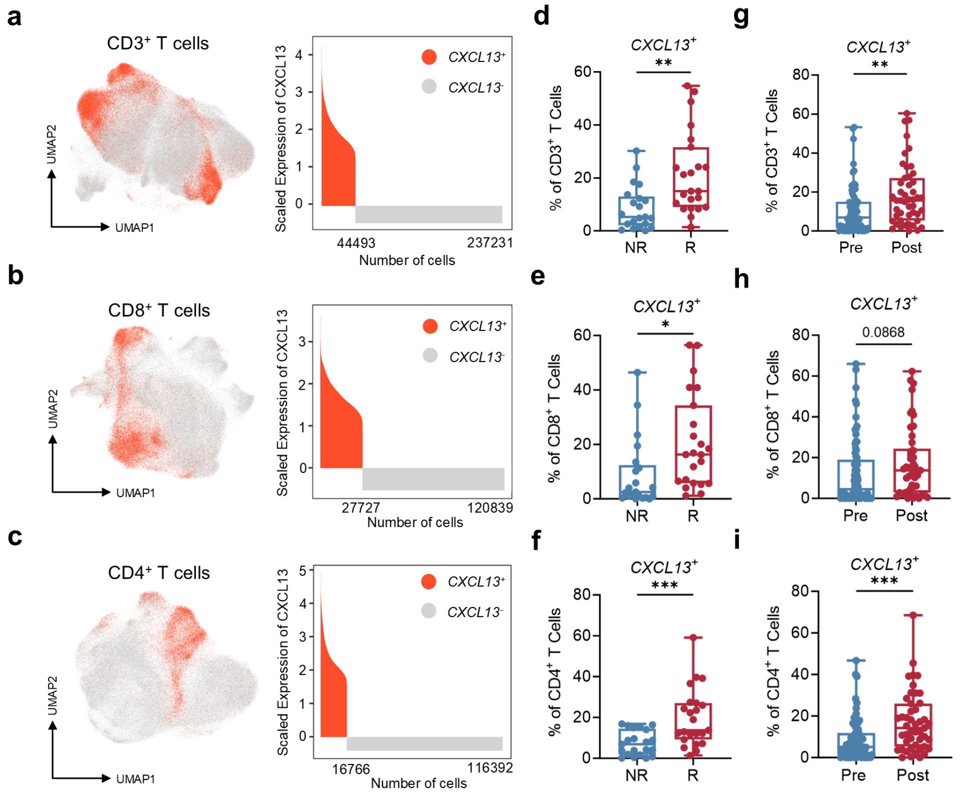

Fig. S2. Distribution and percentage of *CXCL13*$^+$ and *CXCL13*$^-$ cells in ICB treated patients. a-c. UMAP plot and histograms showing the distribution of *CXCL13*$^+$ and *CXCL13*$^-$ cells in CD3$^+$ T cells (a), CD8$^+$ T cells (b), and CD4$^+$ T cells (c). d-i. The percentage of *CXCL13*$^+$ cells in non-response and response groups for CD3$^+$ T cells (d), CD8$^+$ T cells (e), and CD4$^+$ T cells (f). The percentage of *CXCL13*$^+$ cells in pre-treatment and post-treatment groups for CD3$^+$ T cells (g), CD8$^+$ T cells (h), and CD4$^+$ T cells (i). *$p < 0.05$, **$p < 0.01$, ***$p < 0.001$, two-tailed unpaired t test (d-i).

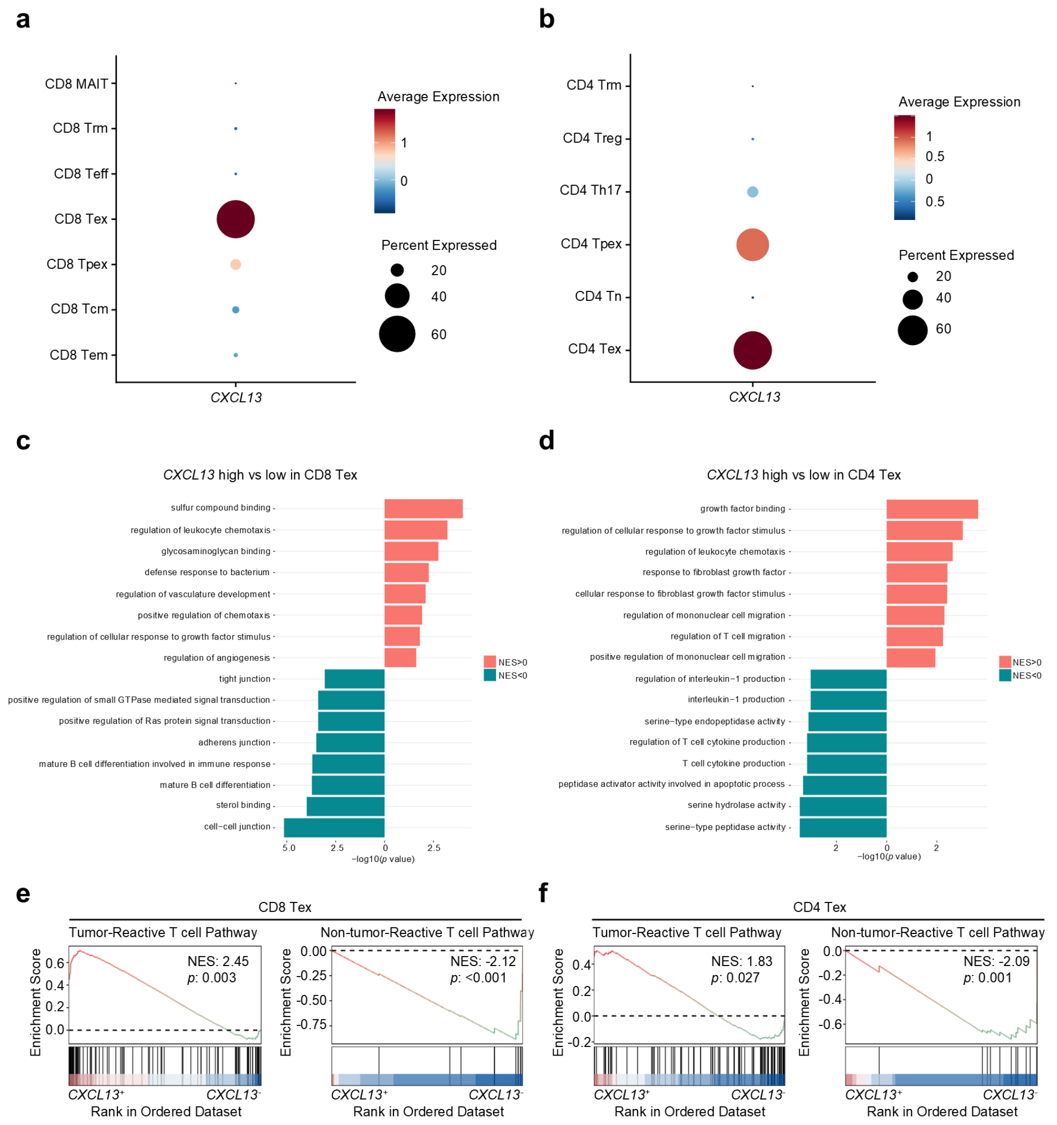

Fig. S3. Expression of CXCL13 in subtypes of CD8 and CD4 T cells, and differential pathways of *CXCL13*⁺ and *CXCL13*⁻ CD8 and CD4 T cells. a-b. Dot plots of *CXCL13* expression in subtypes of CD8 (a) and CD4 (b) T cells. c-d. GSEA of top pathways in *CXCL13*$^+$ vs *CXCL13*$^-$ populations from CD8 Tex (c) and CD4 Tex (d). e-f. GSEA of tumor-reactive T cell pathway and non-tumor-reactive T cell pathway in *CXCL13*$^+$ vs *CXCL13*$^-$ cells in CD8 Tex (e) and CD4 Tex (f).

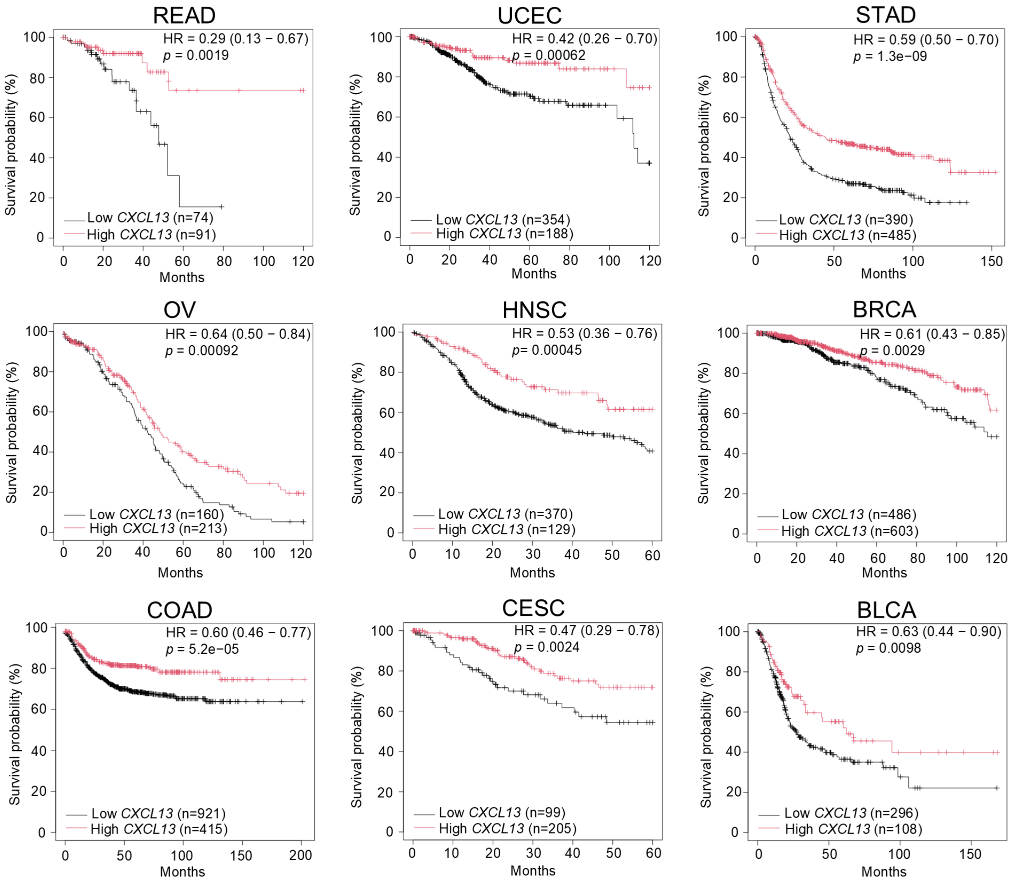

Fig. S4. Overall survival of patients in 9 tumor types based on *CXCL13* expression. Survival probability analysis comparing high *CXCL13* and low *CXCL13* expression groups across the following tumor types using Kaplan-Meier Plotter: READ (Rectum adenocarcinoma), UCEC (Uterine corpus endometrial carcinoma), STAD (Stomach adenocarcinoma), OV (Ovarian cancer), HNSC (Head and neck squamous cell carcinoma), BRCA (Breast invasive carcinoma), COAD (Colon adenocarcinoma), CESC (Cervical squamous cell carcinoma and endocervical adenocarcinoma), and BLCA (Bladder urothelial carcinoma).

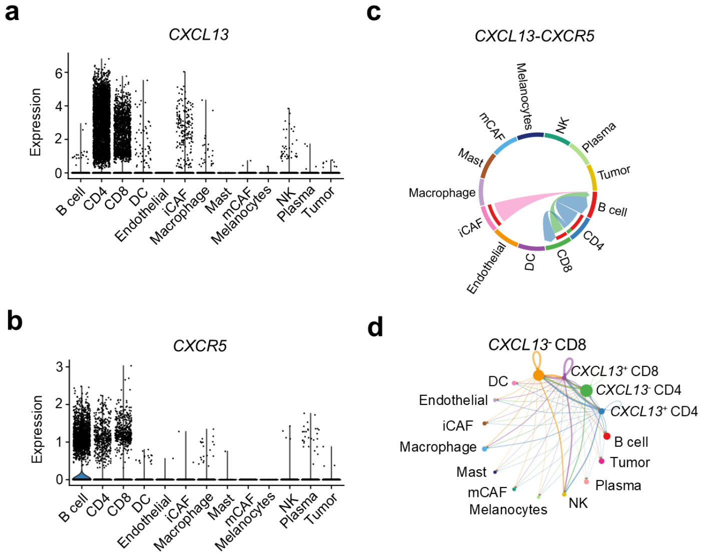

Fig. S5. Expression and cellular communication of *CXCL13* and *CXCR5*. a-b. Expression of *CXCL13* (a) and *CXCR5* (b) in scRNA-seq data from BCC (Basal cell carcinoma). c. Cellular communication of the CXCL13/CXCR5 axis in BCC. d. Cellular communication from *CXCL13*$^{+}$CD4^{+} T, *CXCL13*$^{-}$CD4^{+} T, *CXCL13*$^{+}$CD8^{+} T, and *CXCL13*$^{-}$CD8^{+} T cells, and its effects on all cell types within the tumor microenvironment in BCC.

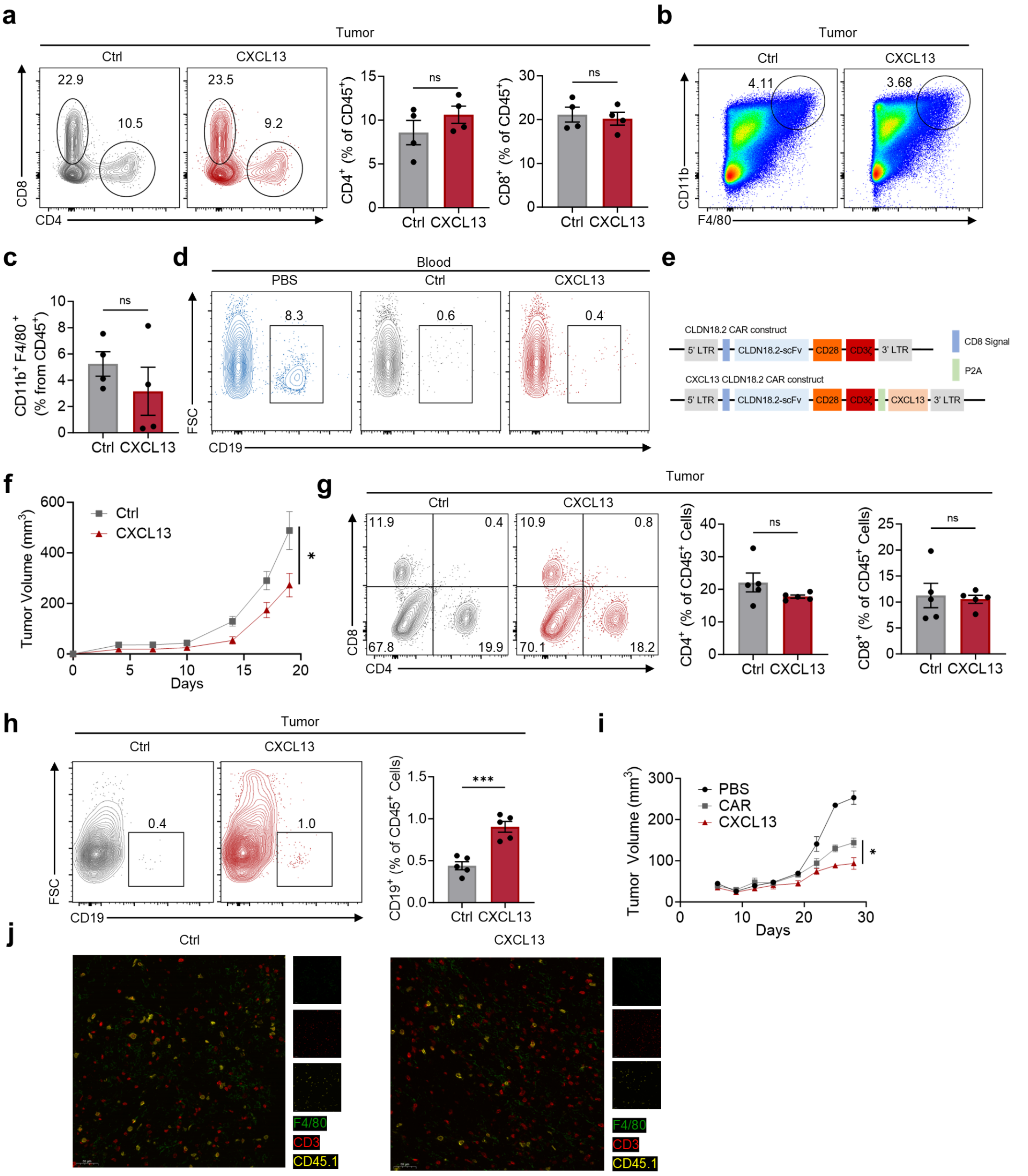

Fig. S6. Analysis of immune cell infiltration and antitumor efficacy of CXCL13 CAR T cells in multiple tumor models. a. Flow cytometry analysis and quantification of endogenous CD4$^+$ and CD8$^+$ T cells in tumors treated with control or CXCL13 CAR T cells. b-c. Flow cytometry analysis of F4/80$^+$ CD11b$^+$ macrophages in CD45$^+$ cells (b) and corresponding quantification (c). d. Flow cytometry analysis of CD19$^+$ B cells in peripheral blood from mice receiving PBS, control CAR T cells, or CXCL13 CAR T cells. e. Schematic representation of CXCL13-overexpressing Claudin 18.2 (CLDN18.2) CAR construct. f-h. C57BL/6

mice injected s.c. with 5×10^5 B16-CLDN18.2 cells were treated with 2×10^6 control or CXCL13 CLDN18.2 CAR T cells on day 5 post-tumor establishment. Tumor growth curves of B16-CLDN18.2-bearing mice (f). Flow cytometry analysis and quantification of endogenous $CD4^+$ and $CD8^+$ T cells in B16-CLDN18.2 tumors (g). Flow cytometry analysis and quantification of $CD19^+$ B cells within $CD45^+$ cells in B16-CLDN18.2 tumors (h). i-j. C57BL/6 mice injected s.c. with 1×10^6 MB49-CD19 cells were treated with 2×10^6 control or CXCL13 CD19 CAR T cells on day 5 post-tumor establishment. Tumor growth curves in the MB49-CD19 tumor (i). Representative immunofluorescence images of tumor sections showing $CD3^+$ T cells, $CD45.1^+$ transferred CAR T cells, and $F4/80^+$ macrophage distribution (j). Mean ± SEM, $n = 4$ (a, c); $n = 5$ (f, g, h); $n = 2$ (i- PBS); $n = 3$ (i-CAR/CXCL13). $*p < 0.05$, $***p < 0.001$, two-tailed unpaired t test (a, c, f, g, h, i).

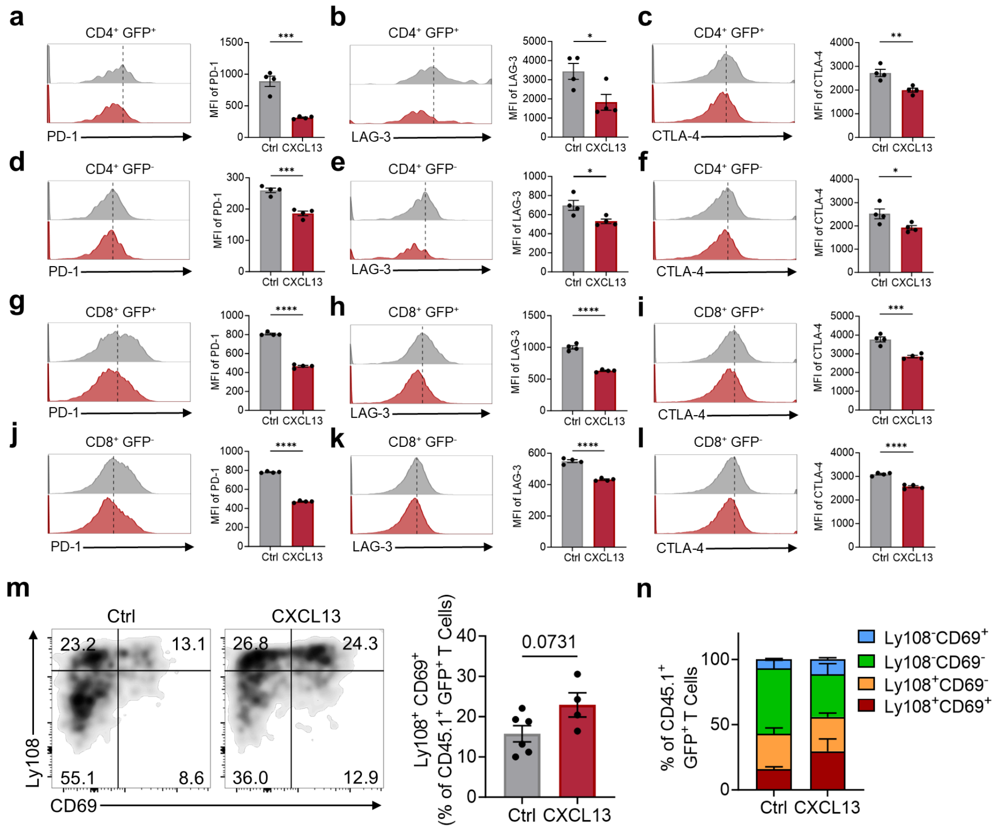

Fig. S7. CXCL13 CAR T cells exhibit reduced exhaustion characteristics. a-l. In vitro expression levels of exhaustion-related proteins, including PD-1 in $CD4^+$ T cells (a, d) and $CD8^+$ T cells (g, j), LAG-3 in $CD4^+$ T cells (b, e) and $CD8^+$ T cells (h, k), and CTLA-4 in $CD4^+$ T cells (c, f) and $CD8^+$ T cells (i, l), in control CAR T cells and CXCL13 CAR T cells. Mean ± SEM, $n = 4$. m-n. Flow cytometric analysis of $Ly108^+CD69^+$CAR T cells (m), and the percentage of cells in the four quadrants (n) in spleen. Mean ± SEM, $n = 6$ (control CAR T), $n = 4$ (CXCL13 CAR T). $*p < 0.05$, $**p < 0.01$, $***p < 0.001$, $****p < 0.0001$; two-tailed unpaired t test (a-m).

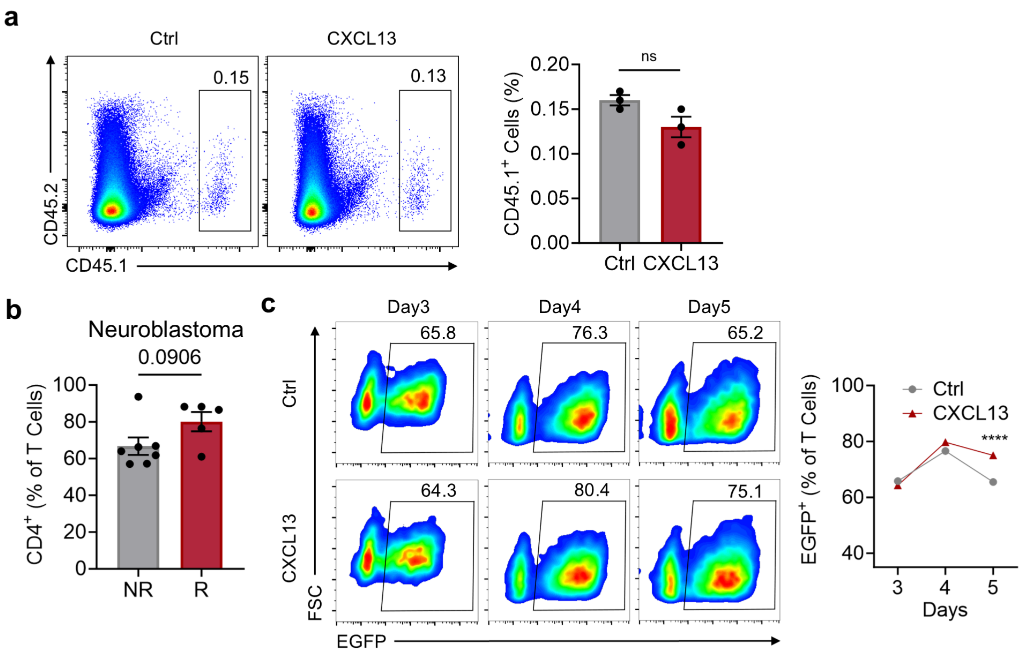

Fig. S8. Proportion of CD45.1$^+$, CD4$^+$ CAR NKT cells and EGFP$^+$ CAR T cells.

a. C57BL/6 mice injected s.c. with $5\text{x}10^5$ B16-CD19 cells were treated with 2×10^6 CAR T cells on day 5 post-tumor establishment. Flow cytometry analysis of the CD45.1$^+$ cells of tumors in control CAR T cells and CXCL13 CAR T cells groups. b. Percentage of CD4$^+$ CAR NKT cells from patients with neuroblastoma treated with CAR NKT cells (non-responder (NR), n = 7 samples; responder (R), n = 5 samples). c. Proportion of EGFP$^+$ CAR T cells in vitro from day 3 to day 5 (n = 4). Mean ± SEM. ****$p < 0.0001$; two-tailed unpaired t test (a, b, c).

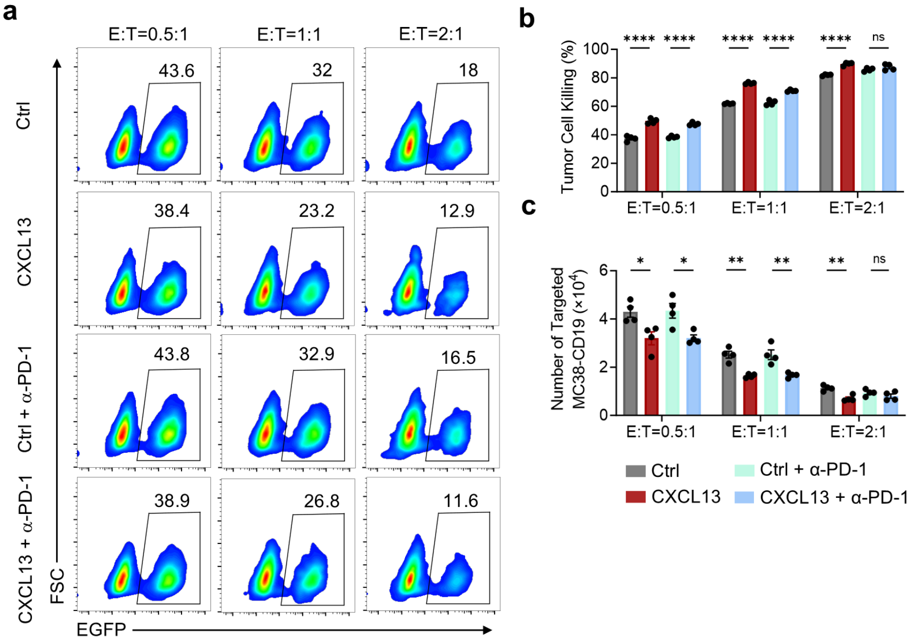

Fig. S9. CAR T cell-mediated tumor cell killing in vitro. a. Co-culture of CAR T cells and tumor cells (MC38 WT [without EGFP]: MC38-CD19 [with EGFP] = 1:1) for 14 hours at different ratios. Percentage of $EGFP^+$ cells in live tumor cells was detected. b. Tumor killing efficiency of CAR T cells. c. Number of MC38-CD19 cells after co-culture with CAR T cells. Mean ± SEM, $n = 4$ (b, c). $^*p < 0.05$, $^{**}p < 0.01$, $^{****}p < 0.0001$, one-way ANOVA and Tukey (b, c).

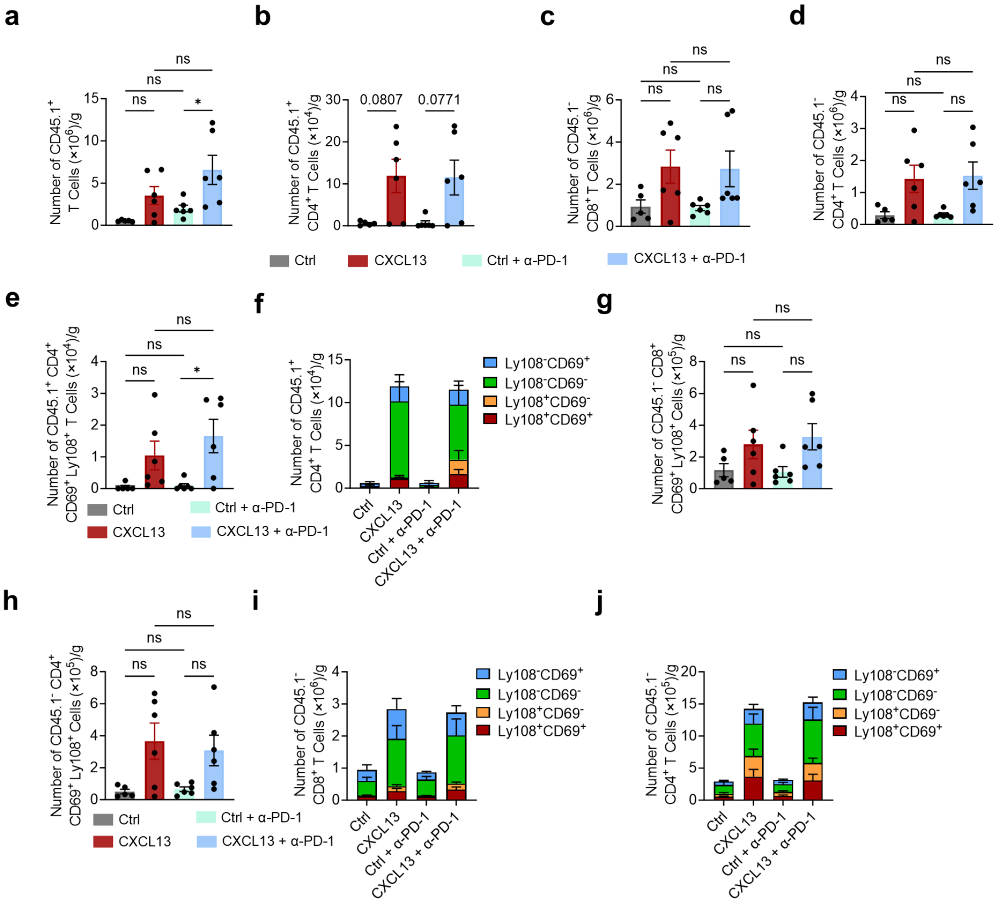

Fig. S10. T cell number and proportion of Ly108$^+$CD69$^+$T cells in tumors. a. Number of CD45.1$^+$ T cells in tumors per gram. b. Number of CD45.1$^+$CD4$^+$ T cells in tumors per gram. c-d. Number of CD45.1$^-$CD8$^+$ (c) and CD45.1$^-$CD4$^+$ (d) T cells in tumors per gram. e. Number of CD45.1$^+$CD4$^+$Ly108$^+$CD69$^+$ T cells in tumors per gram. f. Number of the four stages of exhausted T cells in CD45.1$^+$CD4$^+$ T cells. g-h. Number of CD45.1$^-$CD8$^+$Ly108$^+$CD69$^+$ (g) and CD45.1$^-$CD4$^+$Ly108$^+$CD69$^+$ (h) T cells in tumors per gram. i-j. Number of the four stages of exhausted T cells in CD45.1$^-$CD8$^+$ (i) and CD45.1$^-$CD4$^+$ (j) T cells in tumors per gram. n = 5 (control CAR T), n = 6 (control CAR T + anti-PD-1), n = 6 (CXCL13 CAR T), n = 6 (CXCL13 CAR T + anti-PD-1). Mean ± SEM. *p < 0.05, one-way ANOVA and Tukey (a-e, g, h).
